# Evolving trends in work-family balance research: A bibliometric perspective

**DOI:** 10.1097/MD.0000000000045851

**Published:** 2025-11-14

**Authors:** Yan Yan, Xiaohan Zhang, Chengxin Ye, Jianyi Li, Juan Gao, Yuqing Geng

**Affiliations:** a School of Business, Shanghai Dianji University, Shanghai, China; b Nursing Department, Guizhou Nursing Vocational College, Guizhou, China.

**Keywords:** enrichment, family, gender role, visualization, work

## Abstract

**Background:**

Work-family balance (WFB) is crucial for sustainable development goals and has received considerable attention. However, previous studies have mainly addressed specific aspects of WFB without providing a comprehensive overview. This paper offers new insights into the research foundation, relation, status, and prospects of WFB through bibliometric analysis.

**Methods:**

This study employs bibliometric methods based on data from the Web of Science Core Collection, following the BIBLIO checklist for a comprehensive review. A total of 1390 core articles were selected, and CiteSpace was used for the visual statistical analyses of collaboration, co-citation, and co-occurrence.

**Results:**

Our findings indicate a rising trend in related publications. The collaborative network revealed strong internal connections, emphasizing job satisfaction and the need for supportive WFB measures. Co-citation analysis identifies the job demands-resources theory and conservation of resources theory as key frameworks addressing individual differences and environmental influences on WFB. Co-occurrence analysis showed that low fertility rates among higher education groups correlated with their pursuit of WFB. Gender role differences and work pressure contribute to work-family imbalance, while mental health, time management, and moderating roles also emerge as significant issues. These findings offer new insights for future WFB research: topics will diversify to explore positive work-family interactions for enrichment and sustainability. Cross-national and cross-cultural exchanges are key focuses in this field.

**Value:**

This research is innovative, as it provides a comprehensive view of WFB, identifies emerging trends in practice and research, and establishes a broad knowledge framework to help scholars understand WFB achievements.

## 1. Introduction

Work-family balance (WFB) is essential today, enabling individuals to effectively manage their time and energy between work and family. Achieving this balance promotes personal, familial, and career growth. High satisfaction with both work and family indicated a positive psychological state.^[1]^ WFB offers numerous benefits to individuals, families, enterprises, and society. For individuals, it boosts happiness and life satisfaction, while improving relationships and self-esteem.^[2]^ For families, WFB fosters a harmonious environment that enhances communication among members and elevates the quality of life.^[3]^ For enterprises, WFB helps retain top female talent and enhances female representation in male-dominated management; enterprises value WFB, as enhancing family boundary flexibility boosts employee resilience, promotes work participation, and strengthens core competitiveness.^[4,5]^ From a societal perspective, WFB promotes social welfare, social stability, and gender equality.^[6]^

Currently, many WFB practices are implemented globally. Individuals can balance work and family through effective time management, which involves prioritizing tasks and meticulous planning.^[7]^ They achieve WFB by setting boundaries between work and family, seeking support from family members, sharing household and childcare responsibilities with spouses, and obtaining external assistance when conflicts arise (e.g., domestic services or childcare).^[8,9]^ Many companies help employees balance work and family responsibilities through supportive policies. For example, Google offers 18 weeks of paid maternity and paternity leave, Microsoft has established subsidized childcare centers at its headquarters, IKEA promotes WFB through internal training, and Ernst and Young provides psychological counseling via its employee assistance program. These companies create a supportive environment for WFB, with flexible arrangements and family friendly benefits.^[10,11]^ From a national perspective, Nordic countries like Sweden and Norway excel in gender equality and fathers’ involvement in childcare. In Sweden, parents share 480 days of paid parental leave, 90 days reserved for fathers, contributing to high-quality early childhood education and overall social satisfaction.^[12]^ East Asian countries, such as South Korea, offer free public childcare for children aged 0 to 5,^[13]^ whereas Japan has increased its childcare capacity and encouraged female workforce participation.^[14]^ These individual, organizational, and national practices provide valuable insights and have outstanding significance for WFB.

In theoretical research, the WFB has received considerable scholarly attention. Previous studies have mainly focused on the following aspects. First, studies on influential factors on WFB. Gender differences, unstable income, family responsibilities, and work pressure negatively affected WFB.^[15,16]^ Traditional gender roles often place women as primary caregivers, leading to salary disparities and conflicts due to dual responsibilities.^[17,18]^ Psychological issues, performance-driven workplaces, working overtime, and unequal social resources further worsen this imbalance.^[19–21]^ These challenges are prevalent and remain a key focus of researchers. Second, studies on the mechanisms of WFB in families, organizations, and society. For instance, families can ease child-rearing pressures by utilizing grandparents for free care services,^[22]^ while couples can improve communication and emotional support to tackle life or work challenges.^[23]^ Organizations should prioritize the well-being of their employees by providing emotional support and encouraging managers to implement WFB.^[21,24]^ Fostering inclusiveness through flexible work arrangements can create a conducive environment.^[25]^ At the societal level, promoting paid parental leave and advocating salary equality between genders can create a supportive environment for WFB.^[26]^ Third, there is research on the theoretical foundations of the WFB. For instance, job demands-resource (JD-R) theory examines how resources mitigate burnout from demands,^[27]^ and boundary theory explores how individuals manage boundaries between work and family.^[28]^ Conservation of resources (COR) theory relates to self-leadership strategies in regulating conflicts by safeguarding resources such as time and energy.^[29]^

Therefore, in both management practice and theoretical research, WFB is significant for personal growth, family well-being, and social productivity. While existing studies have addressed themes such as work-family conflict (WFC), supportive measures, satisfaction, psychology, gender differences, and job burnout, notable research gaps remain. Specifically, the absence of visual images illustrating the dynamic cooperative connections of the WFB hinders a comprehensive understanding of cooperative links, current status, and hotspot evolution. Most prior studies have concentrated on social phenomena with detailed observations but lack comprehensive references for future practices or research directions. Additionally, a complete knowledge framework to help scholars grasp the overall picture of WFB is absent.

To address these gaps, this study employed bibliometric methods and the visualization tool CiteSpace to explore 4 research questions:

1. Has this research area received significant attention?
2. What are the key relationships and themes of cooperation in this field?
3. What is the status of the impactful research in this field?
4. What are the emerging hotspots and key trends in the field?

First, we analyzed the statistics of key publications in the WFB field, including annual publication trends, journal metrics, and category analysis. Next, we visualized the collaboration networks among authors, institutions, and regions to highlight important collaborators and themes. Additionally, we examined the co-citations of influential authors, journals, and references to outline the theoretical foundations and current research status of the WFB. We then performed a hot topic analysis using co-occurrence categories, keywords, and bursts to trace the evolution of significant topics in WFB research. Finally, we summarized our findings into a comprehensive knowledge framework highlighting the representative content from each section while identifying hot topics and projecting future practical and research trends.

The structure of this paper is organized as follows. The first section provides a general introduction to the research background and significance. Section 2 outlines the analysis and data selection process. Section 3 presents statistical, cooperation, co-citation, and co-occurrence analyses, which form the core of this study. Section 4 discusses the knowledge framework and future research trends, summarizing key insights and displaying future directions. Section 5 concludes with a summary of the research findings, highlighting the innovations and limitations.

## 2. Methods

This section will follow the BIBLIO checklist guidance steps to construct the bibliometric analysis of WFB, which includes data sources, search strategy, time period, eligibility criteria, data refinement process, quality assessment, and data synthesis.

### 2.1. Data sources

We select the renowned Web of Science Core Collection (WOS CC) as the database for this study. The WOS CC focuses on collecting high-quality articles and providing detailed information on institutions, journals, countries, authors, and citation contexts.^[30]^ It includes 3 core indices and additional extended indices. For literature analysis in WFB, we select the core indexes: Science Citation Index Expanded (SCIE), Social Sciences Citation Index (SSCI), and Arts & Humanities Citation Index (AHCI) because of their rigorous peer review processes. Moreover, this aligns with bibliometric databases commonly used in academia, further demonstrating its universal applicability.^[31,32]^

Therefore, all our data sources were from the SCI/SSCI/AHCI articles in the WOS CC database, excluding other related articles.

### 2.2. Search strategy

We searched using the following query:

1. The topic is “"work*" Near/3 "famil*" Near/3 "*balanc*"” OR “"work*" Near/3 "famil*" Near/3 "*equilibr*".”
2. The document type is “Article,” “Review.”
3. The language is “English.”
4. The publication date is “2000-01-01” to “2024-12-31.”

### 2.3. Time period

This study selected the literature screening period from January 1, 2000 to December 31, 2024. The choice of year 2000 as the starting point was based on our search formula, indicating its optimality in the selected databases. The reasons are as follows: (1) In terms of quantity, the annual number of published papers in the WFB field was low before 2000 but gradually increased after 2000. This indicates that the field has not yet gained significant large-scale attention before 2000. (2) In terms of citation frequency, among the 28 WFB-related papers published before 2000, only 7 were cited more than 30 times, indicating a weak influence. In contrast, of the 8 papers published in 2000 alone, 4 received more than 30 citations. This shows a significant increase in academic attention in this field since the beginning of the 21st century, further validating the choice of using 2000 as a starting point. (3) From a temporal perspective, bibliometric research from 2000 to 2024 spans over 24 years. It comprehensively outlines the foundation, connections, current status, and prospects of the WFB field since the early 21st century. This provides long-term data support for WFB research and enhances its reliability.

### 2.4. Eligibility criteria

Identifying the inclusion and exclusion criteria for articles is very important for selecting the basis for analysis. The inclusion criteria for this study were complete publications in the core database, topic articles that were very close to the WFB theme, and articles from January 1, 2000 to December 31, 2024.

The exclusion criteria were as follows: noncore articles, core articles with low correlation with “work-family balance” retracted articles, selected articles outside the time frame, and articles that did not meet the search criteria.

### 2.5. Data selection process

We collected 1444 articles through the retrieval steps. To ensure relevance and accuracy, we screened the following articles:

1. According to the search strategy, 1444 articles were retrieved and included in the review for subsequent steps.
2. *Thematic relevance review*. The process consisted of 2 reviewers screening for relevance independently, with disputes referred to a third reviewer for resolution. The screening criteria for theme relevance were directly related to the WFB theme. This includes synonyms or interactions such as “work to family balance,” “family to work balance,” “work and family equilibrium,” “work-life balance,” “work-family conflict,” “work-family enrichment,” and “work-family interface.” It also encompasses core studies on elements like “satisfaction,” “imbalance,” “role,” “gender role,” etc. They can also be balancing measures related to the family or work. In conclusion, the criterion for assessing topic relevance is the provision of references for the WFB bibliometrics. Consequently, 52 irrelevant articles were excluded, leaving 1392 relevant articles.
3. *Full-text assessment*. To verify the completeness of the 1392 articles and check for any retractions, prefaces, withdrawals, or data integrity issues, we conducted a full-text review. Ultimately, 2 retracted articles were identified and excluded, resulting in 1390 qualified articles.
4. *Duplicate removal*. We exported 1390 articles from WOS in “full text” format as input data for CiteSpace to perform deduplication. After the “CiteSpace: Duplicates Removal” step, we found that there were no duplicate records, and all 1390 articles were recognized by CiteSpace, confirming its effectiveness.
5. In the end, we selected 1390 qualified articles.

**Figure 1.**
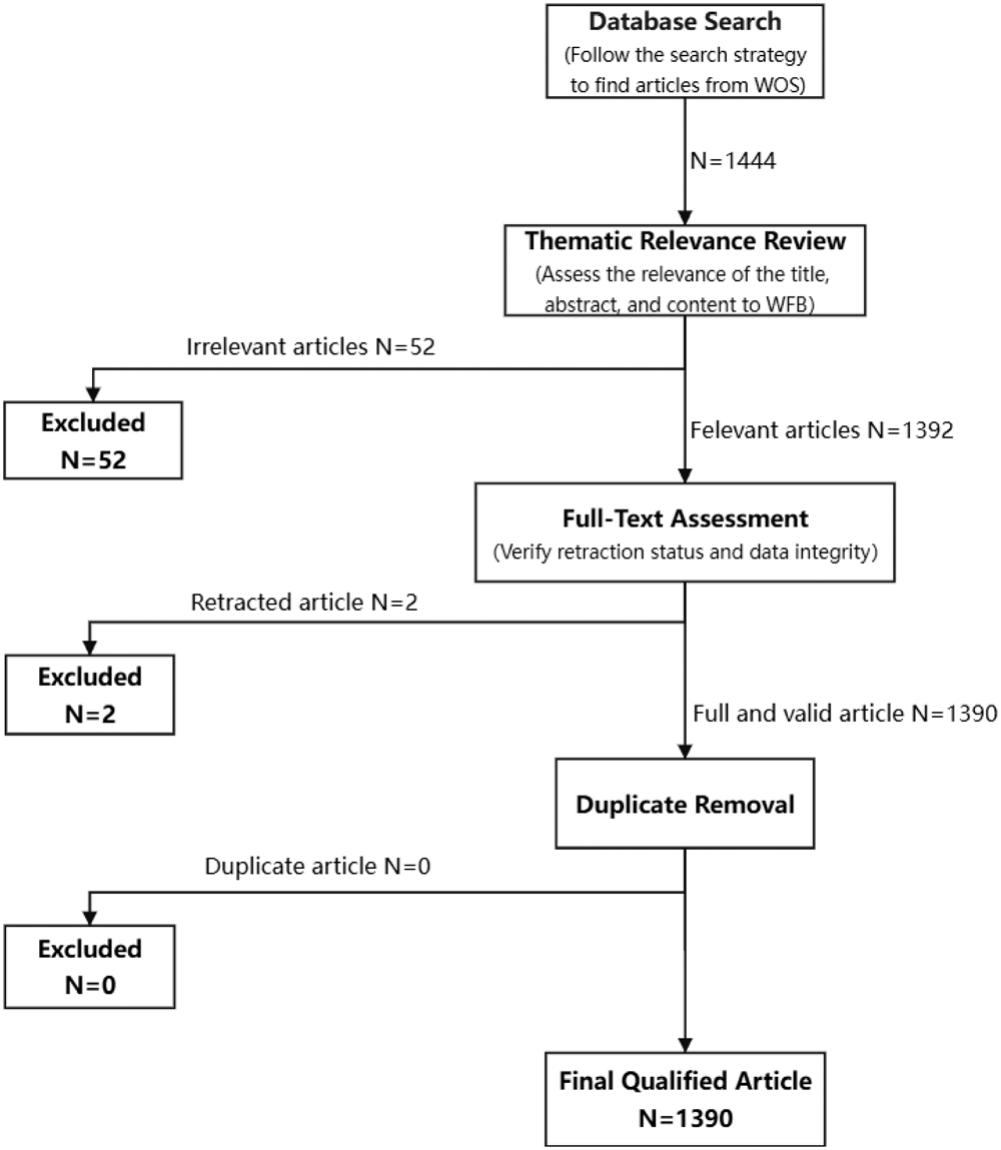
Flowchart of data selection process. WFB = work-family balance, WOS = Web of Science.

The flowchart is in Figure 1.

### 2.6. Quality assessment

We performed a quality assessment using the BIBLIO checklist to reduce any possible risk of bias and created a standard table (Table 1). The approach involved 3 authors independently evaluating 1390 articles using a quality assessment table. The evaluation focused on 3 dimensions:

**Table 1:** Quality assessment criteria.

Evaluation dimensions	Evaluation criteria	Scope of the article	Assessment
Theme relevance	1. The literature addresses work-family balance. Such as “work-family balance,” “work to family balance,” “family to work balance,” “work and family equilibrium,” “work-life balance,” “work-family conflict,” “work-family enrichment,” “work-family interface,” etc. 2. The literature highlights key elements of work-family dynamics, including “satisfaction,” “imbalance,” “role,” “gender role,” “well-being,” “family roles,” “traditional gender division of labor,” and “organizational support,” etc. 3. The literature explores the balance in a single dimension of work or family, such as career development versus work stress, work stress management, distribution of family responsibilities, and child care. 4. The literature explores work-family coordination issues in specific contexts, such as the negative impact of work pressure on medical staff’s families, family satisfaction among remote workers, and the psychological experiences of working parents during the epidemic.	1390 articles	Qualified
	1. The article fails to meet the 4 standards mentioned above. 2. It only addresses a single aspect of “work,” “family,” or “balance.”	1390 articles	Unqualified
Data integrity	1. Complete full text, including essential information like research background, methods, and results. 2. Exclude “retract,” “withdraw,” or “preface” literature. 3. Include only non-duplicated literature.	1390 articles	Qualified
	Does not meet the above standards.	1390 articles	Unqualified
Source and type standardization	1. From the SSCI/SCI/AHCI databases of WOS CC. 2. Document types: “Article” or “Review.” 3. Language: English. 4. Publication date: January 1, 2000 to December 31, 2024.	1390 articles	Qualified
	Does not meet the above standards.	1390 articles	Unqualified

AHCI = Arts & Humanities Citation Index, SCI = Science Citation Index, SSCI = Social Sciences Citation Index, WOS CC = Web of Science Core Collection.

1. *Theme relevance*: Literature is qualified if it explicitly addresses “work-family balance,” discusses key elements between work and family, explores balance at the individual or family level, or examines coordination in specific groups. It is unqualified if it does not meet these criteria or focuses solely on one aspect of “work,” “family,” or “balance.”
2. *Data integrity*: A document is qualified if it has complete text and is not categorized as “retract,” “withdraw,” “preface,” or a duplicate; otherwise, it is unqualified.
3. *Source and type standardization*: Qualified articles must be from SSCI/SCI/AHCI databases, classified as “articles” or “reviews,” written in English, and published between January 1, 2000 and December 31, 2024. Articles that did not meet these criteria were considered unqualified.

Disagreements on the evaluations were settled by the fourth author through a review of the original literature and group discussions.

Ultimately, the author independently verified that all 1390 articles met the criteria across all 3 dimensions, ensuring the reliability of the subsequent analyses.

### 2.7. Data synthesis

This study utilized the CiteSpace 6.3. R1 for analysis, objectively extracting statistical data on authors, institutions, categories, and keywords (encompassing quantity, centrality, and starting year) and visually mapping clustering relationships.^[33]^ This tool enables us to perform quantitative and qualitative analyses on the clustering themes of each part, establishing a knowledge framework based on “foundation - relation - status - prospect.” The research themes, methods, and disciplinary attributes identified will serve as key references for future trends, highlighting significant directions in this field. The WFB research maintains these core attributes by moving forward. Specifically, we conducted the following analysis using CiteSpace.

1. Publication statistical analysis relies on the annual publication volume, journal count, and categories from the WOS interface. These data were utilized to generate figures and tables for quantitative analysis and to summarize the publication trends and disciplinary attributes of WFB research qualitatively. This section outlines the WFB’s publication trends, examines the popularity of its topics, and forms the foundation for the entire text. This addresses the first question: Has this research area gained significant attention?
2. In the collaboration analysis, CiteSpace was used to visualize collaborations among authors, institutions, and regions. Key collaborators in the WFB field can be identified based on their frequency and centrality. Extract clustering labels with the LLR algorithm, and analyze key collaborative themes, research directions of institutions, and cross-regional achievements. They addressed the question: What are the key relationships and collaboration themes in this field? This is a relational section of the text.
3. Co-citation analysis employs CiteSpace to visually examine authors, journals, and references. The timeline illustrates the co-citation relationships among authors, indicating the dynamic changes within the same clustering theme. Additionally, data statistics and visual clustering were used to analyze the co-citation relationships between journals and references, highlighting key achievements and foundational themes. Co-citation analysis highlights the significant research status in this field and addresses the third question: What is the status of impactful research here?
4. CiteSpace was employed for in-depth analyses of co-occurrence categories, keywords, and keyword bursts. Co-occurrence analysis revealed core themes within each category, evolution of keyword hotspots, and their sudden emergence through cluster analysis, timelines, and keyword bursts. These insights guide future research by highlighting emerging hotspots and key trends in the field, thus forming the prospect section of the text. This section answers the fourth question: what are the emerging hotspots and key trends in this field?

Ultimately, we created a comprehensive knowledge framework from 4 parts, emphasizing the literature characteristics in the WFB field through the “foundation – relation - status - prospect.” This framework highlights the significance of the study. Future research should comprehensively consider the above analysis results for each part by integrating their interrelated situations. For example, the interdisciplinary attributes in the category publication analysis and findings in the category co-occurrence section mutually confirm each other, reinforcing the core conclusion of interdisciplinary integration. Similar clustering themes across different sections also highlight the key research areas in this field.

**Figure 2.**
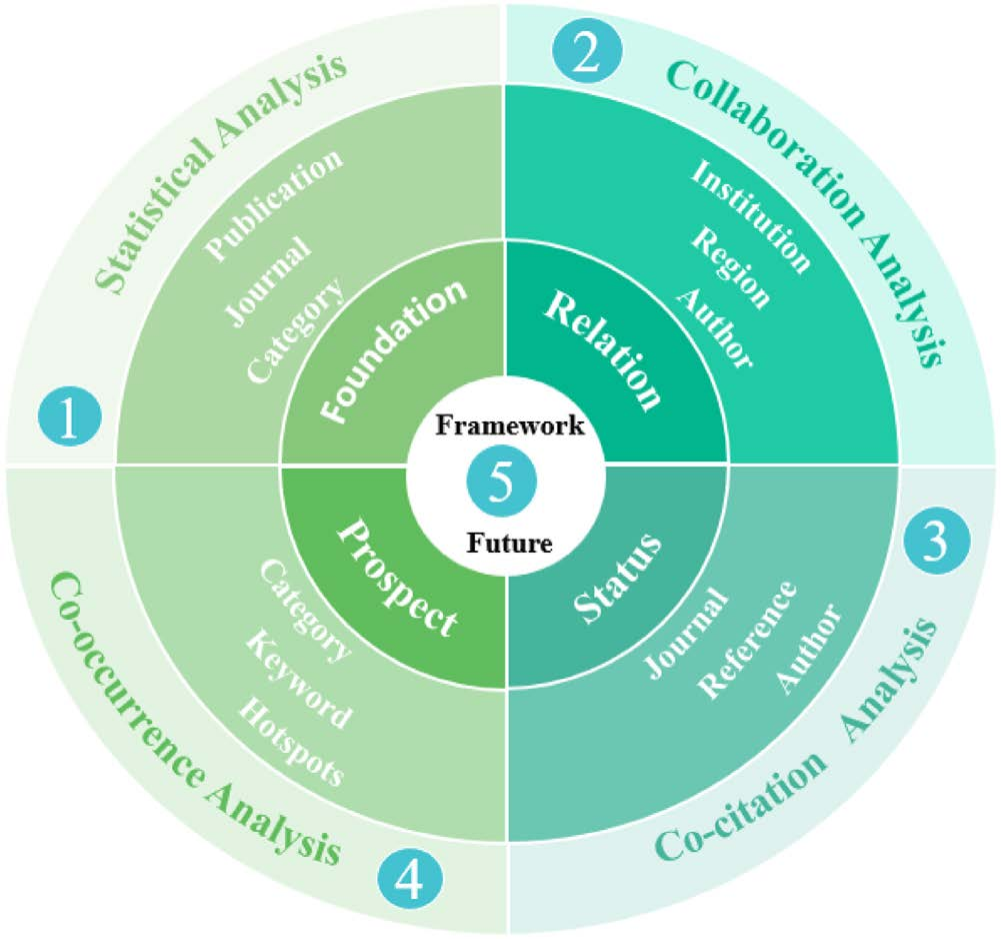
Study steps.

The research steps are illustrated in Figure 2.

## 3. Results

### 3.1. Publication statistical analysis

Statistical analysis of publications assesses research by year, journal, and category quantitatively. This overview highlights the trends in WFB research and forms the foundation for the entire paper, which addresses the first question "Has this research area gained significant attention?"

#### 3.1.1. Annual publications trends

**Figure 3.**
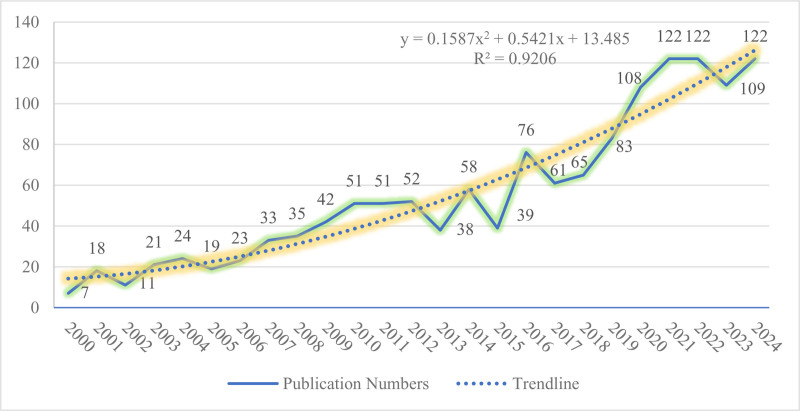
Rising trend in work-family balance field publications.

Figure 3 illustrates the annual volume and trends in WFB research, which can be divided into 3 stages.

1. The first stage (2000–2012) shows steady growth, with articles increasing from 7 in 2000 to 52 in 2012. One of the earliest studies highlights differing pressures on full-time versus part-time working women, revealing that part-time workers experience less stress but lower life satisfaction,^[34]^ the topic still relevant today. This stage also featured diverse findings. For instance, Bygren and Duvander examined how fathers’ work status affects parental leave usage,^[35]^ while Sayer and Fine explored racial differences in work-family responsibilities among married White and Black women.^[36]^ The second stage (2013–2018) was marked by fluctuations. The number of articles rose from 38 in 2013 to 58 in 2014 but fell back to 39 in 2015. In contrast, it surged to 76 in 2016 before declining again. This instability may reflect shifts in the research focus. The third phase (2019–2024) sees explosive growth peaking at 3 points: 2021, 2022, and 2024, all with 122 papers, 17 times more than the initial phase’s output of 7 papers (2000), indicating a surge in WFB’s popularity among researchers. Highly cited works during this period indicate that COVID-19 significantly influenced interest in WFB research, including flexible working arrangements, gender equality, economic pressure, children’s education, and family labor.^[37–39]^
2. We also employed regression analysis to create a trend line that illustrates the increasing research on WFB. The trend line formula is *y* = 0.1587*x*^2^ + 0.5421*x* + 13.485, where “*y*” represents the number of publications per year and “*x*” denotes the year. As years progress, the number of annual publications is expected to increase. The fitting index *R*^2^ = 0.9206, which is close to 1, indicates high prediction reliability.

Overall, articles on WFB have steadily increased and are expected to continue to rise, underscoring the need for further comprehensive analysis.

#### 3.1.2. Journal publication analysis

Over the past 24 years, 1390 articles on WFB have been published in 200 journals, with 41 journals featuring 6 or more articles. This finding indicates a diverse range of publications. Table 2 lists the top 14 journals by publication volume, along with their 5-year impact factor (IF), journal citation reports (indicating relative standing within the discipline), and percentage of the total journal output. The key findings are as follows:

**Table 2:** Statistical publications journals.

Ranking	Journal	Count	5-Year impact factor	Quartile	Percentage
1	*Frontiers in Psychology*	32	3.3	Q2	2.302%
2	*Journal of Marriage and Family*	29	5.1	Q1	2.086%
3	*Journal of Family Issues*	28	2.1	Q2	2.014%
4	*International Journal of Environmental Research and Public Health*	25	4.8	Q2	1.799%
5	*Journal of Vocational Behavior*	25	9.4	Q1	1.799%
6	*International Journal of Human Resource Management*	20	5.8	Q1	1.439%
7	*Sustainability*	20	3.6	Q2	1.439%
8	*Work: A Journal of Prevention Assessment Rehabilitation*	16	1.9	Q3	1.151%
9	*Journal of Family Studies*	14	1.6	Q3	1.007%
10	*Journal of Family and Economic Issues*	12	2.8	Q2	0.863%
11	*Journal of Managerial Psychology*	12	4.5	Q2	0.863%
12	*PLoS One*	12	3.3	Q1	0.863%
13	*Sex Roles*	12	4.2	Q1	0.863%
14	*Work Employment and Society*	12	4.8	Q1	0.863%

1. Psychological and family studies, along with work and socioeconomic journals, frequently publish articles on WFB. Keywords: *Frontiers in Psychology* (32 articles, 2.302%), *Journal of Marriage and Family* (29 articles, 2.086%), *Journal of Family Issues* (28 articles, 2.014%), *Journal of Family Studies* (14 articles, 1.007%), *Sex Roles* (12 articles, 0.863%). These journals explore topics from both the psychological and familial perspectives.^[40–42]^ In contrast, the *Journal of Vocational Behavior* (25 articles, 1.799%), the *International Journal of Human Resource Management* (20 articles, 1.439%), *Work: A Journal of Prevention Assessment Rehabilitation* (16 articles, 1.151%), and the *Journal of Family and Economic Issues* (12 articles, 0.863%) examined WFB through career development and economic pressures.^[43,44]^
2. Most journals in this field were ranked Q1 and Q2, with IFs of 1 to 6, indicating the prevalence of high-quality publications. The *Journal of Vocational Behavior* stands out, with a notable 5-year IF of 9.4. The journal’s most cited topics were quality of life, workplace culture, supportive spouses, emotional expression, and sleep quality.^[45,46]^ Studying articles in the *Journal of Vocational Behavior* provides insights into quality research on WFB.
3. The authors’ submission criteria should consider various factors. For example, the *International Journal of Environmental Research and Public Health* has a Q2 ranking and an IF of 4.8. However, it was removed from the WOS core journal catalog because of inconsistent quality and academic misconduct. This suggests that authors should assess not only a journal’s ranking and IF but also its publication volume and quality control standards.

Based on this analysis, researchers can submit papers to psychological and family studies journals, as well as work and economic relations journals. We also recommend that they consider journal reputation, quality, and topic relevance rather than relying solely on partitions and IF to ensure that their research has a lasting impact.

#### 3.1.3. Category publication analysis

The subject category analysis revealed the distribution of subjects in the WFB research. Table 3 lists the top 11 categories.

**Table 3:** Statistical publications categories.

Ranking	Category	Count	Percentage
1	Management	196	14.173%
2	Public, Environmental & Occupational Health	172	12.374%
3	Psychology Applied	150	10.791%
4	Family Studies	144	10.360%
5	Sociology	110	7.914%
6	Psychology Multidisciplinary	94	6.763%
7	Women's Studies	71	5.108%
8	Social Sciences Interdisciplinary	69	4.964%
9	Economics	60	4.317%
10	Business	59	4.245%
11	Industrial Relations Labor	59	4.245%

1. WFB studies were primarily associated with “Management” and “Public Environmental Occupational Health,” comprising 369 articles (26.547% of the total).
2. Two key disciplines from psychology include “Psychology Applied” (150 articles, 10.791%) and “Psychology Multidisciplinary” (94 articles, 6.763%), underscoring the importance of psychology in this area.
3. Four social sciences categories accounted for 394 articles, representing 28.345% of relevant literature: “Sociology” (110 articles), “Family Studies” (144 articles), “Women’s Studies” (71 articles), and “Interdisciplinary Social Sciences” (69 articles). “Business” and “Economics” also contribute significantly.

This analysis highlights the interdisciplinary nature of management, psychology, social sciences, and business in WFB research, which promotes collaboration among scholars. Specifically, management studies focus on industrial relationships, work-family boundary management, working time management, and the impact of parental status on managers. For instance, some scholars have studied how leadership behavior affects employee turnover and found that perceived peer support moderates the relationship between leadership behavior and work-family spillover, thus reducing turnover rates.^[47]^ Some scholars have explored how female entrepreneurs in patriarchal societies can manage their entrepreneurial and familial roles better through boundary management strategies. They found that nonconfrontational negotiation methods, particularly the integration of work and family, can help minimize conflicts.^[48]^ Some scholars have found that, in male-dominated work environments and modesty-valuing cultures, job security, job quality, and interpersonal relationships are more likely to enhance women’s job satisfaction than salary and promotion opportunities.^[49]^ In addition, WFB from the psychology discipline examines an individual’s subjective experiences, including happiness, satisfaction, burnout, self-compassion, stress, and enthusiasm in both work and family. It also explores how internal emotions and external stressors affect the balance between work and family through psychological coping measures. For instance, some psychologists have found that self-compassion is more effective than rumination, self-esteem, and mindfulness in reducing WFC from the perspective of positive psychology.^[50]^ They also found that young people with positive parent-child relationships are more likely to lose confidence in their WFB self-efficacy when they witness conflicts between their parents’ work and family lives.^[51]^ Some scholars, from a relational perspective, draw on cognitive and affective processing systems and COR theory to clarify the relational influences of work and family on WFB self-efficacy and find that family social resources are more effective than work support in alleviating WFC.^[52]^ Social sciences mainly focus on universal social issues, such as low fertility rates caused by factors such as economic pressure, gender inequality, and policy deficiencies; the work-family situations of special occupational groups (such as medical staff, internet platform workers, migrant workers, miners, etc); the stress of female workers during the pandemic; and paternity leave and child-rearing subsidy policies. Economics and business examine new working patterns, entrepreneurial mothers, and organizational performance. For example, some scholars have explored how unemployment or immigration can encourage mothers’ entrepreneurial behavior. At the same time, fathers’ paternity leave serves as crucial support for mothers when starting a business.^[53]^ Some scholars have examined how human resource management practices influence employees’ creative performance. They found that better communication between team leaders and members strengthens the indirect effect of employees’ Psychological capital on creativity. This suggests that leaders should prioritize communication with employees to improve organizational performance and achieve greater benefits.^[54]^ In conclusion, various disciplines emphasize different aspects. Future scholars should enhance the theme of WFB by considering the unique research focus of each discipline.

#### 3.1.4. Summary

In conclusion, annual publication trends indicate a rising interest in WFB, suggesting that it will continue to attract attention. Journal publication analysis revealed that journals that focus on psychology, family studies, and economics are key areas for selection. Future scholars should consider factors such as reputation, quality, and sustainability when choosing journals to mitigate risk. The statistical analysis shows that knowledge in WFB primarily encompasses management, psychology, and business economics. Future research should draw from theories in these core disciplines to enrich WFB knowledge and focus closely on related fields to improve objectivity and accuracy.

### 3.2. Cooperation characteristics analysis

This section examines the collaboration among authors, institutions, and regions in the WFB field. Its aim is to clarify collaboration relations, assess cooperation impacts, analyze results, and offer recommendations for future partnerships. It also sought to address the second question: What are the key relationships and collaboration themes in this field?

#### 3.2.1. Author collaboration

Table 4 presents the top 8 authors of the most collaborative studies on WFB, globally. Overall, collaboration among authors in this field was limited, with only 22 out of 651 coauthors collaborating more than twice, indicating a weak sense of connection.

**Table 4:** Top 8 collaborative authors in WFB field.

No.	Count	Centrality	Year	Authors
1	7	0.00	2012	Tammy D Allen
2	5	0.00	2021	Berta Schnettler
3	5	0.00	2016	Toyin Ajibade Adisa
4	5	0.00	2021	Ligia Orellana
5	4	0.00	2008	Lyn Craig
6	4	0.00	2021	Clementina Hueche
7	4	0.00	2016	K Michele Kacmar
8	4	0.00	2016	Maria Jose Chambel

WFB = work-family balance.

1. The author with the highest number of collaborations was Tammy D Allen (7 times), suggesting his impact in this area. He provided an example for Berta Schnettler, Toyin Ajibade Adisa, and Ligia Orellana (all 5 times). Their cooperation spans different directions; for instance, Tammy D Allen offers 4 interpretations of the WFB theory (2-way conflict and abundance, low conflict and high abundance, balance satisfaction, and balance effectiveness) while exploring their impact on job performance and family satisfaction.^[55]^ Additionally, he has conducted studies on transnational couples’ WFB types and teachers’ time allocation between work and family.^[56,57]^ Berta Schnettler and Ligia Orellana focus on working families with adolescent children,^[58]^ whereas Toyin Ajibade Adisa specializes in semi-structured interviews to examine single parents’ WFB. His research investigates the imbalance faced by single student-working mothers compared to single self-employed parents^[59,60]^
2. The first author to collaborate was Lyn Craig (4 times, 2008), who established a model for future partnerships. His early coauthored articles examined parents’ experiences with WFB, the impact of family friendly workplaces on satisfaction, and how flexible schedules can enhance work-family satisfaction for mothers of adolescents.^[61]^ In summary, collaboration among authors creates an excellent opportunity to explore diverse research in WFB. Recognizing these key collaborators boosts confidence in subsequent researchers and aids in selecting suitable partners.

**Figure 4.**
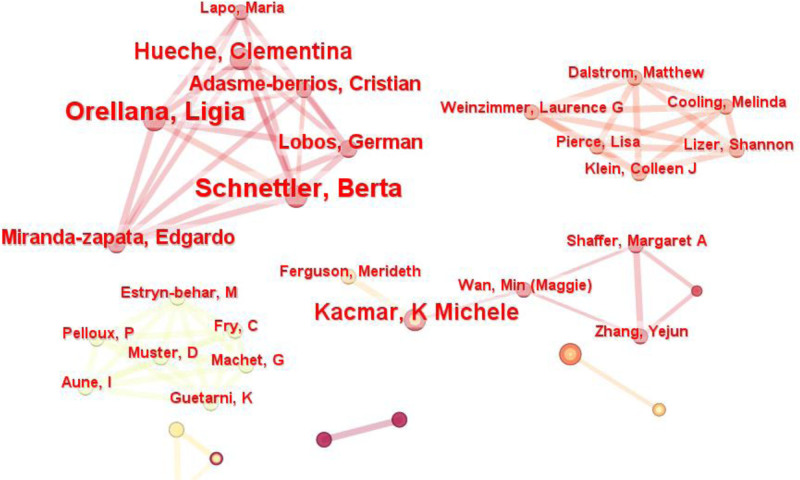
Network of collaboration authors (node = 653; link = 378).

Figure 4 illustrates a clustering diagram of the author’s collaboration network. The diagram revealed relatively independent collaborative groups with close internal relationships, highlighting 4 significant research teams.

1. Ligia Orellana and Berta Schnettler collaborated extensively and had numerous coauthors. Their research focused on the link between family satisfaction and WFB, indicating that lower family satisfaction may correlate with reduced family income, increased parental depression and anxiety, and work-family imbalance.^[62,63]^
2. The team led by Michele Kacmar examined WFB in dual-earner families. They found that supportive spouses with work-related connections could foster common topics and mitigate stress transmission, thereby reducing marital tension and enhancing family satisfaction.^[64]^ Additionally, a harmonious workplace and work enthusiasm contribute to achieving WFB.^[65]^
3. Matthew Dalstrom’s team aimed to enhance the work input of advanced practice providers by distributing questionnaires and employing structural equation modeling. Their study revealed that work stress from job demands and the work environment negatively affected work engagement. However, WFB, clear role division, and autonomy could alleviate stress responses and boost engagement.^[66,67]^
4. Estryn-Behar’s team investigated the relationship between doctors’ job burnout and WFC. Their findings showed that turnover intention and burnout are common among French doctors, with family factors playing a significant role. Female emergency physicians often experience burnout owing to childcare responsibilities.^[68]^ Additionally, issues such as WFC, effort-reward imbalance, and poor teamwork quality contribute to this challenge.^[69]^ In conclusion, while coauthors in the field of WFB have diverse research interests, there is a notable lack of collaboration among them. Building more coauthor networks is crucial for sharing academic resources and enriching research outputs.

#### 3.2.2. Institution collaboration

Table 5 highlights the top 10 agencies that frequently collaborate in WFB research. Understanding these partnerships can reveal valuable research directions. The details are as follows:

**Table 5:** Top 10 collaborative institutions in WFB field.

Ranking	Institution	Region	Count	Centrality	Year
1	University of California System	USA	28	0.04	2002
2	State University System of Florida	USA	26	0.07	2001
3	Harvard University	USA	23	0.13	2001
4	California State University System	USA	20	0.12	2001
5	University of Texas System	USA	19	0.05	2007
6	University System of Ohio	USA	16	0.07	2002
7	Pennsylvania Commonwealth System of Higher Education (PCSHE)	USA	14	0.08	2004
8	University of London	UK	14	0.16	2002
9	La Trobe University	Australia	13	0.00	2008
10	University of Quebec	Canada	12	0.01	2011

WFB = work-family balance.

1. American institutions dominate collaboration, with the top 7 from the United States establishing early partnerships. Notably, the University of California System has been the most collaborative, conducting several studies on workplace low back pain linked to work-family imbalance. They believe that this imbalance causes work pressure and adversely affects their physical health.^[70,71]^ The State University System of Florida, Harvard University, and California State University System were early pioneers in this field post-2000. Their primary focus was on how organizational support improves WFB by enhancing employee commitment, motivation, satisfaction, and happiness.^[72]^
2. The University of London (14 articles, 0.16) had the highest centrality, indicating its leadership in this field. Collaborative research from this institution aids in identifying cutting-edge directions. For instance, it recently partnered with Zhejiang University to explore family motivation as a potential stressor for work-related stress and insomnia, recommending physical activity, and family incentives to alleviate such stress.^[73]^ Harvard University also exhibited significant centrality, with a score of 0.13. It focused on WFB within the medical system with Johns Hopkins University, revealing that female doctors often prioritize child-rearing over career advancement.^[74]^
3. La Trobe University in Australia and the University of Quebec in Canada began their collaboration relatively late (2008 and 2011, respectively) with low centrality (almost 0). This indicates a brief period of research on WFB, highlighting the potential for growth and the need for stronger academic partnerships to enhance its impact. Recently, La Trobe partnered with the University of Western Australia to study how Vietnamese immigrants manage childcare stress, revealing that most prefer unpaid care from their parents due to high costs.^[22]^ In 2023, the University of Quebec collaborated with various institutions to investigate the “double-edged sword” effect of remote consultation on WFB and gender differences in satisfaction regarding this balance.^[75–77]^

Research shows that developed countries in Europe and the United States excel. Developing countries should prioritize WFB, improve interagency cooperation, and seek diverse solutions.

**Figure 5.**
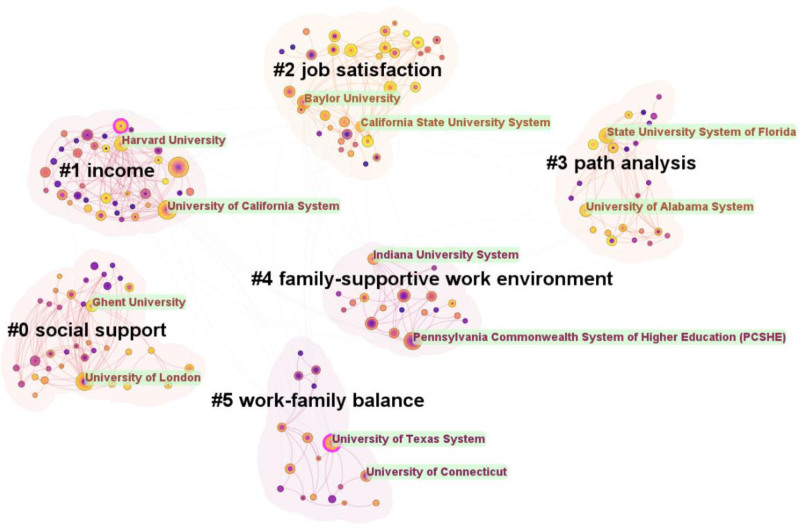
Cluster graph of institutions cooperation (node = 463; link = 574).

Figure 5 illustrates the visual cluster diagram of institutional collaboration comprising 463 nodes and 574 links. Each cluster label indicates a collaboration topic, whereas the node labels identify the top 2 collaborating institutions within each cluster. The analysis is as follows:

1. Some clusters focused on promoting WFB. For instance, cluster #0 (social support) includes Ghent University and the University of London as key partners. Collaborative articles in this cluster emphasized social support tools for WFB, such as flexible working arrangements and emotional encouragement at home. Examples include flexible parental leave for parents of seriously ill children, and practical advice to enhance social support systems.^[78]^ These measures can positively impact WFB. Cluster #4 (family supportive work environment) highlights how family support enhances the work atmosphere, leading employees to feel more grateful and responsible for their families. The Indiana University System and Pennsylvania Commonwealth System of Higher Education collaborate with Sungkyunkwan University to explore how supportive work environments enable employees to meet family responsibilities. They recommend that organizations implement paid leave policies to foster family friendly workplaces and strengthen their emotional connections with employees.^[79]^
2. Some clusters examined the factors affecting the WFB. For example, cluster #5 (work-family balance) focuses on the core theoretical application of WFB, such as the COR theory guiding individuals to acquire resources in work, family, and society to help them achieve WFB, in collaboration with key institutions such as the University of Texas System and the University of Connecticut. Research by the University of Texas System indicates that WFC generates negative emotions that impact WFB satisfaction; however, resources from both domains can alleviate this conflict and enhance overall satisfaction.^[52,80]^ Cluster #2 (job satisfaction) connects job satisfaction to WFB. Baylor University and the California State University System conducted research showing that mindfulness improves job satisfaction by accelerating resource accumulation and enhancing happiness, while addressing poor WFB.^[81]^
3. Some clusters focus on various aspects of the WFB field. For instance, cluster #1 (income), represented by the University of California System and Harvard University, examines the income gap due to gender differences across industries. A collaborative study by Harvard indicates that women in senior positions in large international firms face significant disparities in promotion and income compared to men, mainly because they are expected to shoulder more family responsibilities.^[82]^ Additionally, research with the Rothman Orthopaedic Institute revealed that female orthopedic surgeons in the United States earn an average of $100,000 less annually than their male counterparts.^[83]^ Cluster #3 (path analysis) examines how factors such as work-family mutual penetration, education, family friendly policies, and race influence WFB through path analysis. The State University System of Florida and University of Alabama System represent this cluster. This study adopts a bioecological framework perspective to explore how work positively impacts family life, and how family harmony enhances work performance. It also analyzes the bridging effect of work-family interpenetration on WFB in terms of nonstandard working hours (such as overtime, shift, etc) and relationship quality (such as the harmonious degree of the parent-child relationship).^[84]^

In summary, different institutions prioritize varied research areas. We recommend that future research entities seek collaborators aligned with their specific interests to foster knowledge integration and enhance academic contributions in this field.

#### 3.2.3. Region collaboration

Table 6 lists the top 10 regions for WFB collaboration, detailing the number of collaborations, network centrality, and the year each collaboration started. Notably, 9 are developed countries. The details are as follows:

**Table 6:** Top 10 collaborative regions in WFB field.

Ranking	Region	Count	Centrality	Year
1	USA	489	0.05	2000
2	Peoples R China	135	0.04	2005
3	Australia	109	0.12	2002
4	Canada	100	0.09	2000
5	England	95	0.52	2002
6	Spain	79	0.00	2008
7	Germany	55	0.29	2007
8	South Korea	49	0.00	2008
9	Netherlands	46	0.07	2000
10	Italy	43	0.01	2007

WFB = work-family balance.

1. The United States has led 489 regional collaboration since 2000, indicating its leadership in WFB issues supported by ongoing research. China ranks second among developing countries with 135 collaborations since 2005 but needs to strengthen its influence. Recent studies indicate that increasing mechanization in rural China may provide farmers more time for their children’s education.^[85]^
2. England had the highest centrality at 0.52, indicating its core position and influence in the regional cooperation network. Germany and Australia follow with centralities of 0.29 and 0.12, respectively, reflecting their influence within the network. These figures underscore the key role of these 3 countries in regional collaboration.
3. The earliest collaborative areas were the United States, Canada, and the Netherlands, which began collaborative research in 2000 due to their unique social contexts. For example, early discussions in the United States focused on workplace discrimination related to family responsibilities and emphasized WFB.^[86]^ In Canada, a diverse economy has fostered many female entrepreneurs; thus, balancing work and family while achieving self-worth has become a key research topic.^[87]^ Dutch scholars studied how rapid economic development disrupts work-family dynamics and recommended enhancing welfare benefits for affected employees.^[88,89]^

Emphasis on employee family responsibilities and supportive cultures in these regions establishes a foundation for research on WFB.

In conclusion, countries with robust human rights protections emphasize WFB. The United States is a research leader, China is an important developing partner, and the United Kingdom is a notable influencer. This trend provides valuable insight for future international collaborative research.

**Figure 6.**
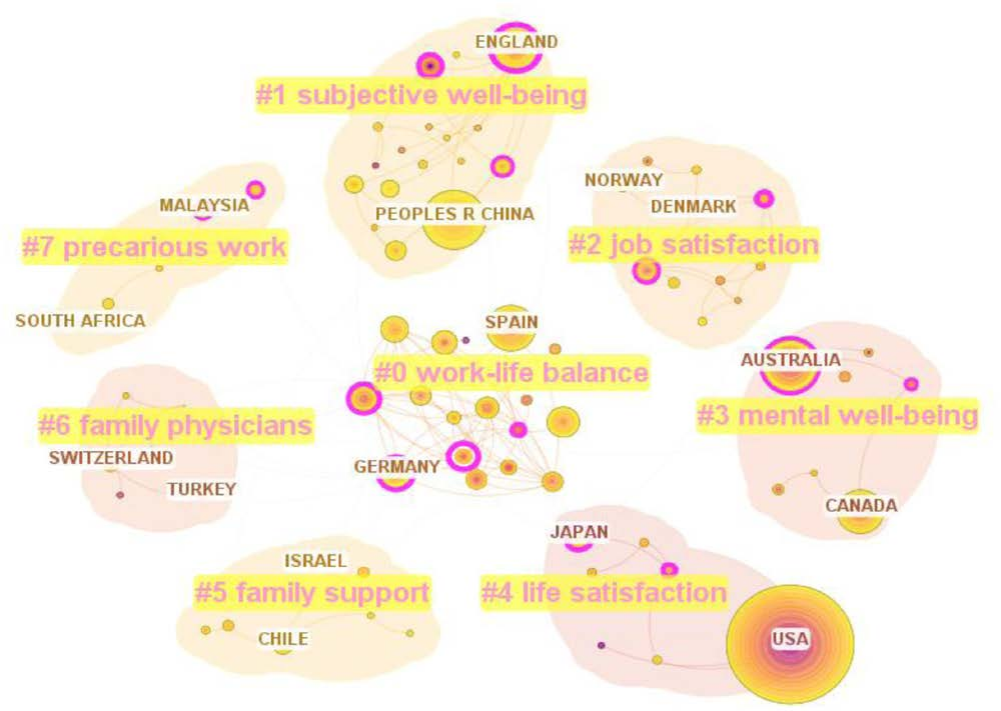
Cluster graph of regions cooperation (node = 82; link = 141).

Figure 6 shows the regional collaborative cluster diagram. We use a tree-ring history to illustrate node shapes, reflecting changes in collaborative countries over time. The purple outer ring indicates high intermediary centrality, whereas the red inner ring represents recent active collaborations. Details are as follows:

1. Some clusters focused on personal well-being and psychological aspects of WFB, including #1 (subjective well-being), #2 (job satisfaction), #3 (mental well-being), and #4 (life satisfaction). England and China represent cluster #1, which emphasizes individuals’ subjective perceptions and assessments of their quality of life. This cluster serves as a significant psychological indicator for evaluating the effectiveness of the WFB. It investigates the various factors that influence happiness and overall satisfaction, such as work arrangements and gender roles. Chinese researchers evaluate subjective well-being through happiness, life satisfaction, health status, and depression levels, finding that involuntary overtime significantly negatively affects well-being.^[20]^ Cluster #2 (job satisfaction) refers to an individual’s assessment of their job. Low job satisfaction can lead to negative emotions that affect family life, resulting in work-family imbalances. Conversely, high job satisfaction indicates resource replenishment from work and can benefit the family. For example, in cluster #2, Norwegian studies on dairy farmers revealed that automated milking systems, systematic training, and better communication enhanced job satisfaction related to WFB.^[90]^ Cluster #3 examined the mental health of workers in certain countries. Australian research links mental ill health with WFC, and suggests a positive correlation between mental health and life satisfaction. They noted that work stress can lead to anxiety and depression.^[91,92]^ Cluster #4 (life satisfaction) assesses an individual’s subjective view of their overall well-being. Life satisfaction is a broader evaluation than job or family satisfaction, covering areas such as work, family, and personal development. Cluster #4 includes the United States and Japan. Recent US studies highlight the benefits for parents achieving WFB: improved parenting quality, reduced stress/conflict, and increased life satisfaction.^[93]^
2. Some clusters focused on family and social support to improve WFB, such as #5 (family support) and #6 (family physicians). Cluster #5 (Family support) refers to leveraging family resources to enhance WFB, such as kinship networks, spousal assistance, and emotional support, which can create a positive feedback loop for WFB. Representative nodes for #5 are Israel and Chile, with Chile collaborating with Ecuador. Based on COR and role balance theories, they found that dual-earner parents leverage family resources to enhance life satisfaction through family support.^[94]^ Cluster #6 (family physicians) focuses on parents who serve as primary caregivers in the family. They often struggle with work-family imbalance due to long-term care for sick children or spouses, leading to hidden working hours and emotional exhaustion. Providing assistance to them is crucial. Representative nodes for #6 were Switzerland and Turkey. In Turkey, researchers partnered with the United States to tackle WFB issues faced by mothers of children with autism, who often have a negative outlook on work and life while acting as “family physicians.” They recommended providing these mothers with emotional support and legal training.^[95]^
3. There are clusters focusing on broader subject categories and work characteristics, such as #0 (work-life balance) and #7 (precarious work). Cluster #0 covers a broader theme than the WFB, with representative nodes in Spain and Germany. Work-life balance refers to the equilibrium between work, family life, friends, hobbies, and other aspects.^[96]^ A joint study by Spain and the United Kingdom found that high managerial autonomy positively influences family life satisfaction through improved home performance.^[97]^ Cluster #7 (precarious work) highlights the characteristics of precarious work, which lacks stability and essential labor protection. This directly erodes the economic foundation of the WFB by systematically depriving it of resources. A joint study by Malaysia and Brunei found that compared to decent work, precarious work is significantly limited in employment stability, social dialogue participation, and workers’ rights protection. It was also noted that precarious work increases the risk of work-family imbalance.^[98]^

It is essential to note the significant difference in centrality between the representative countries of cluster #1 in Figure 6: the United Kingdom has a distinct purple circle around its node, while China does not. This indicates that China lags behind the United Kingdom in terms of collaboration influence. However, according to Table 6, China ranks second in collaboration, following the United States. The difference between Figure 6 and Table 6 indicates that China’s collaboration is more quantitative than qualitatively impactful. Therefore, China should enhance its collaboration with various regions to strengthen its core influence in collaboration networks.

In summary, cross-collaborative research can be conducted in various regions. We recommend that future scholars focus on regional influence and collaboration, select regions with a strong willingness to collaborate, and engage in cross-regional research.

#### 3.2.4. Summary

In summary, the analysis explored author collaboration, institutional collaboration, regional collaboration, and thematic trends. The most active authors were led by Ligia Orellana and Schnettler. The University of California System showed the highest willingness to collaborate. Simultaneously, the United States was the leading region in collaboration. In addition, different collaborative groups focus on various themes, such as work-family satisfaction, stress, family support, and mental health. Future discussions will continue on these topics, with an emphasis on expanding cross-regional and cross-cultural cooperation to enhance the WFB research outcomes.

### 3.3. Co-citation characteristics analysis

Co-citation analysis revealed the contributions of co-cited authors, journals, and references in the field of WFB. By identifying highly co-cited content, we can outline evolving research hotspots and deepen our understanding of WFB. The cluster labels represent common topics in articles referencing these co-cited works, reflecting the current research focus. This addresses the third question: what is the status of impactful research in this field?

#### 3.3.1. Author co-citation

Co-citation of authors refers to the occurrence of 2 or more authors being cited together in a publication. Table 7 lists the top 10 co-cited authors with at least 128 citations, highlighting their significant reference value in this field.

**Table 7:** Top 10 author co-citation in WFB field.

Ranking	Author	Count	Centrality	Year
1	JH Greenhaus	448	0.02	2000
2	TD Allen	280	0.01	2004
3	MR Frone	256	0.03	2001
4	DS Carlson	255	0.03	2001
5	EE Kossek	242	0.03	2002
6	JG Grzywacz	230	0.01	2004
7	P Voydanoff	156	0.07	2001
8	AB Bakker	142	0.05	2010
9	RG Netemeyer	137	0.06	2001
10	SE Hobfoll	128	0.03	2010

WFB = work-family balance.

1. JH Greenhaus had the highest number of co-citations (448) and was first cited in 2000, indicating his early focus on this research area. He specializes in work-family role penetration and employs meta-analysis to explore the factors affecting WFB.^[99,100]^ His recent highly cited articles define “balance” as a psychological perception based on employees’ satisfaction evaluations regarding their work and family roles. This understanding stems from their emotional experiences and effectiveness in fulfilling responsibilities within these roles,^[101]^ offering subsequent scholars a more precise grasp of “balance.”
2. Voydanoff is a highly co-cited author (156 times, 0.07, 2001), indicating a significant influence of citations with various authors. His key article shows that family demands negatively impact work performance, whereas family resources can enhance it.^[102]^ His research covers the work-family interface, role performance in both domains, work demands and resources, and WFC.^[103]^ These contributions are foundational for future scholars to explore the relationship between work and family dynamics.
3. AB Bakker and SE Hobfoll have frequently been cited together since 2010, reflecting their significant contributions to research over the past decades. Bakker, a highly cited psychologist, examined JD-R theory, which suggests that job resources (such as social support and career development) and personal resources (such as optimism and self-efficacy) can reduce employee burnout from job demands (e.g., workload and time pressure).^[104,105]^ He advocates using this theory to analyze WFC for better WFB.^[106]^ Hobfoll introduced COR, emphasizing that individuals have limited resources (time, energy, emotions) to manage work-family imbalances. He highlighted the importance of resource replenishment through priority adjustments, rest, and seeking support^[107]^ with subsequent scholars widely accepting his theory.

In summary, research on co-cited authors is essential in this field. The analysis aids scholars in understanding the key contributions of influential authors and enhances their understanding of the WFB.

**Figure 7.**
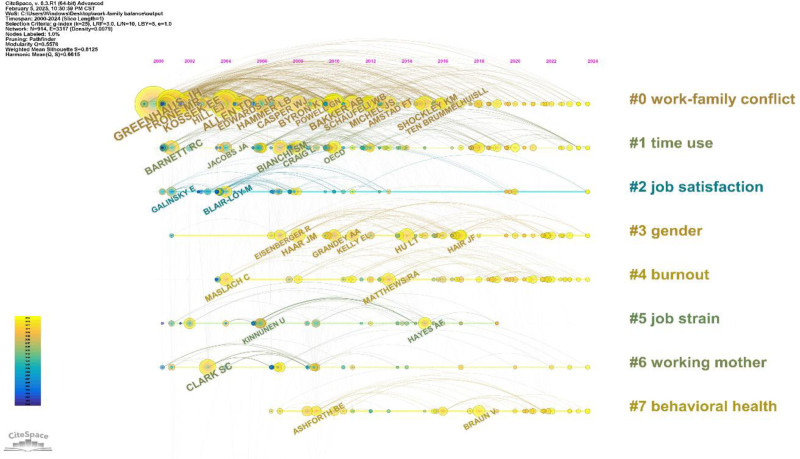
Author co-citation dynamics (node = 914; link = 3317).

We created a timeline of author co-citations (Fig. 7) to show the evolution of key co-citation authors across topics. Node size reflects citation frequency and academic influence, whereas lines indicate co-citation relationships between authors.

1. Clusters #0 (work-family conflict), #1 (time use), #2 (job satisfaction), #3 (gender), and #6 (working mothers) have been significant since 2000 and will continue until 2024. The co-cited authors #0 and #1 are closely related. Cluster #0 (work-family conflict) focuses on disputes arising from incompatible work and family role demands. These factors include mutual interference between work and family,^[108]^ cultural differences, resource depletion,^[109]^ and uneven distribution of personal resources.^[110]^ Self-compassion and emotional self-control may help to reduce these conflicts.^[50]^ Cluster #1 (time use) in the WFB context highlights differences in time and effort among individuals across work-family modules, emphasizing the role of time management in WFB. RC Barnett, a key co-cited author, explored stress from work-family time conflicts, such as the clash between parents’ work hours and children’s school schedules, which causes anxiety for working parents.^[111]^ The authors also noted that female employees may have lower turnover rates if reduced working hours improve their marital quality and satisfaction with motherhood.^[112]^ Cluster #2 (job satisfaction) examined how workplace discrimination and family responsibilities influence job satisfaction. Blair-Loy, another prominent co-cited author, studied gender imbalance and found that high-intensity jobs reduce women’s roles as caregivers, leading to decreased job satisfaction after childbirth.^[113]^ Clusters #3 (gender) and #6 (working mother) explored gender dynamics in the WFB. The workplace’s gendered structure and division of labor at home lead to distinct WFB experiences and strategies for men and women. For instance, Grandey focused on differences in gender norms; he argued that women’s public displays of pride often receive less recognition than men’s similar behaviors. This highlights why men and women approach WFB differently.^[114]^ These long timelines indicate that more co-cited authors have studied this clustering label topic, underscoring its ongoing relevance in WFB.
2. Authors in cluster #4 (burnout) and #7 (behavioral health) have been co-cited recently, indicating a rising trend. Cluster #4 examines burnout as a chronic issue impacting work and home life, emphasizing that workplace interactions, such as civility and respect, affect employee burnout levels,^[115,116]^ indicating that the quality of interpersonal interactions (civility, respect) can act as a work resource to buffer against burnout caused by high demands, which is in line with the JD-R theory. Cluster #7 focuses on healthy behaviors that promote WFB, including a positive emotional tone and mental distance from colleagues.^[117,118]^ Additionally, co-cited author BE Ashforth explored how organizational identity shapes individual behavior. He finds that individuals who identify with their organizations show higher commitment and participation. Conversely, those with weak organizational identification may feel alienated and exhibit increased turnover tendencies, influencing their WFB choices.^[119]^

To summarize, the key topics in this field include WFC, time use, job satisfaction, gender, and mothers in the workplace. Burnout and health behaviors are prominent research areas. We recommend collaboration among co-cited authors with similar interests to enhance the content quality.

#### 3.3.2. Journal co-citation

Table 8 lists the top 10 journals with over 300 citations, categorized by subject according to the Journal Citation Indicator. The details are as follows:

**Table 8:** Top 10 journal co-citation in WFB field.

Ranking	Journal	Count	Centrality	Year	5-Year Impact Factor
1	*Journal of Applied Psychology*	638	0.02	2001	11.2
2	*Journal of Vocational Behavior*	598	0.03	2000	5.8
3	*Journal of Marriage and Family*	494	0.01	2001	14.7
4	*Academy of Management Review*	470	0.05	2000	17.1
5	*Journal of Organizational Behavior*	403	0.03	2001	11.2
6	*Human Relations*	378	0.03	2000	12
7	*Journal of Management*	342	0.02	2001	7
8	*Journal of Occupational Health Psychology*	339	0.03	2006	11.2
9	*Journal of Family and Economic Issues*	329	0.03	2000	10.1
10	*Academy of Management Journal*	300	0.05	2000	6.3

WFB = work-family balance.

1. The *Journal of Applied Psychology* had the highest citation count (638 citations, center degree 0.02). It examines how cultural factors impact employee psychology and organizational behavior,^[120,121]^ positioning itself within management and applied psychology. As the most co-cited journal in this field, it enhances the understanding of how gender, personality, occupation, and lifestyle influence WFB through strategies for organizational insight, positive image maintenance, and emotional stability.^[122]^ It was found that exhaustion, stress, and emotional backlash lead to WFC.^[123]^
2. The centrality of the top 10 co-cited journals ranges from 0.01 to 0.05, indicating a lack of significant network leaders in this field, with all showing average influence. The *Academy of Management Review* and the *Academy of Management Journal* had the highest centrality at 0.05, establishing them as leading management journals. A notable article in the *Academy of Management Review* explored how personal strategies impact work-family satisfaction, demonstrating that adjusting resources and barriers, allocating resources, prioritizing goals, and modifying objectives can effectively manage the work-family interface.^[124]^
3. The *Journal of Occupational Health Psychology* ranks 8th, noted for its innovative and impactful content that quickly gains recognition. Recent research has emphasized organizational well-being, individual mental health, and employee safety^[125,126]^ offering more insights into employee mental health and work performance within the WFB context.
4. The IF of the top 10 co-citation journals were all above 5, with 7 exceeding 10, indicating a positive correlation between co-citation and IFs. Notably, the *Academy of Management Review* has the highest IF at 17.1, focusing on business and management. A highly cited article suggests that achieving WFB involves identifying obstacles and using deadlines to prioritize goals.^[124]^ The *Journal of Marriage and Family* also has an IF of 14.7, focusing on parental relationships, family care, and well-being.^[127]^ It is recommended that caring for elderly relatives after marriage should be prioritized during rest rather than after work.^[128]^

This reveals the structure and main research directions of these highly co-cited journals, providing a reference for researchers in selecting journals.

**Figure 8.**
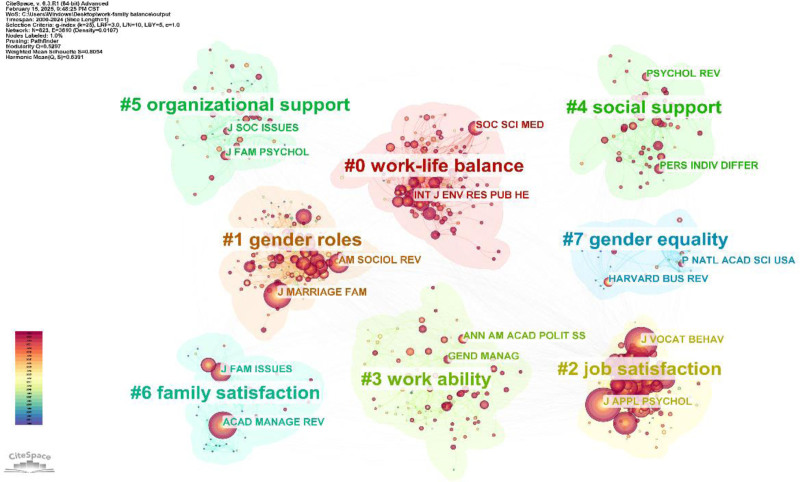
Cluster graph of journals co-citation (node = 823; link = 3610).

Figure 8 presents the journal co-citation clustering diagram, which emphasizes the core topics. The analysis is as follows:

1. Some clusters emphasize the routes to WFB and personal capability factors, such as #0 (work-family balance) and #3 (workability). Cluster #0 combines insights from organizational behavior and health science, indicating ways to achieve balance in work-family boundaries. A recent social science and medicine study found that a healthy work environment has a positive effect on mental health and job satisfaction, and recommended a healthy work environment to mitigate burnout.^[129]^ Cluster #3 focuses on how individual agencies can overcome economic constraints and discusses the role of high employee work ability in achieving this balance. For example, *The Annals of The American Academy of Political and Social Science* reported that higher education levels enable individuals to secure skilled jobs and improve their families’ living conditions.^[130]^
2. Some clusters highlight external factors that promote WFB, such as #4 (social support) and #5 (organizational support). Cluster #4 highlights the importance of social support for achieving WFB, including family oriented assistance from organizations, colleagues, and superiors (404, 2022). *Personality and Individual Differences* note that middle-aged female employees face WFC and lower job satisfaction, while emotional, material, and companionship support from family, friends, and community can enhance resilience and reduce stress (an effective way to achieve WFB).^[131]^ Cluster #5 highlights the benefits of organizational support for WFB. *The Journal of Social Issues* advocates policies such as paid leave and childcare subsidies to alleviate workplace pressure on women by reducing childcare costs.^[132]^
3. Clusters addressing gender role differences and WFB included #1 (gender roles) and #7 (gender equality). Cluster #1 explores how traditional gender roles shape perceptions of responsibility, with the *American Sociological Review* noting that women’s perceived family responsibilities may reduce their career durability compared to that of men.^[133,134]^ The *Journal of Marriage and Family* highlights disparities in labor division, showing that mothers take on more daily household tasks while fathers focus on financial duties.^[135]^ Cluster #7 emphasizes gender equality, advocates for equal treatment of men and women in the workplace, uniform standards regarding family responsibilities, and protection from penalties or added pressure due to gender differences. Its representative journal, *Harvard Business Review*, promotes training to reduce gender bias and creates diverse workplaces^[136]^ aiming for organizational gender equality.^[137]^ However, the *Proceedings of the National Academy of Sciences* points out that achieving this goal is challenging and reports a slowdown in progress toward gender equality in the United States.^[138]^ These references are essential for understanding gender differences in WFB research.
4. Cluster studies focused on #2 (job satisfaction) and #6 (family satisfaction), reflecting individuals’ perceptions of WFB and providing a direct evaluation. Cluster #2 emphasizes enhancing job satisfaction, with the *Journal of Vocational Behavior*, noting that flexibility in work time and location can improve job satisfaction by reducing work-life conflict.^[139]^ The article also explores work enrichment theory and the effort-reward imbalance model to suggest strategies for boosting job satisfaction, such as aligning effort with rewards and minimizing household distractions.^[57]^ Cluster #6 examined factors that enhance family satisfaction. The *Journal of Family Issues* highlights that lower financial pressure, marital happiness, infant health, and social support for single mothers significantly increases family satisfaction.^[140]^ Additionally, couples who shared parenting and earning responsibilities reported higher levels of family satisfaction.^[141]^

To summarize, the key themes in this field include factors that enhance WFB, gender role differences and equality, job satisfaction, and family satisfaction. These themes are prevalent in leading journals, and represent important research topics and trends. These findings offer significant reference values for future studies.

#### 3.3.3. Reference co-citation

Co-citation occurs when later studies cite 2 or more references. Table 9 lists the top 11 references with their total citation counts followed by a specific analysis.

**Table 9:** Top 11 reference co-citation in WFB field.

Ranking	Count	Centrality	Year	Reference
1	39	0.04	2021	V Braun, 2021, QUAL RES PSYCHOL, V18, P328
2	38	0.07	2017	JH Wayne, 2017, PERS PSYCHOL, V70, P167
3	26	0.26	2018	JF Hair, 2018, MULTIVARIATE DATA AN, V0, P0
4	26	0.05	2018	WJ Casper, 2018, J APPL PSYCHOL, V103, P182
5	26	0.08	2006	JH Greenhaus, 2006, ACAD MANAGE REV, V31, P72
6	19	0.04	2005	LT Eby, 2005, J VOCAT BEHAV, V66, P124
7	17	0.01	2005	K Byron, 2005, J VOCAT BEHAV, V67, P169
8	16	0.07	2017	AF Hayes, 2017, INTRO MEDIATION MODE, V0, P0
9	14	0.02	2011	JS Michel, 2011, J ORGAN BEHAV, V32, P689
10	14	0.03	2021	L Craig, 2021, GENDER WORK ORGAN, V28, P66
11	14	0.02	2020	H Chung, 2020, SOC INDIC RES, V151, P365

WFB = work-family balance.

1. The most cited paper was by V. Braun, published in *Qualitative Research in Psychology* in 2021, with 39 citations and a centrality of 0.04. It discusses a topic analysis method for qualitatively analyzing open data to identify meaningful topics, leading to clear reports.^[142]^ Subsequent research using this method explored the factors affecting WFB across various contexts. For example, after semi-structured interviews, some studies conducted a thematic analysis of the impact of work-family boundaries on nurses during COVID-19, identifying viral infectiousness and health anxiety as key factors.^[143]^ Other literature surveyed working mothers about their job and family status, emphasizing job satisfaction and social support. Childcare assistance from employers, colleagues, and family boosts job satisfaction and improves WFB.^[144]^
2. The most central literature was published by JF Hair in the *Journal of Multivariate Analysis* in 2018. It received 26 citations and had a centrality degree of 0.26, indicating high quality and influence. This study introduces partial least squares structural equation modeling (PLS-SEM), which studies variable relationships and is widely used in WFB research. For example, some studies have used PLS-SEM to explore the relationship between work demands (e.g., hours and pressure), family demands (e.g., responsibilities and childcare), work resources (e.g., autonomy and support from superiors), national cultural background, and WFB. They found that both work and family demands were negatively correlated with balance. Additionally, parental status in countries such as France and Italy influences the balance.^[145]^ Other literature employed PLS-SEM to examine connections among WFB, technology compatibility, remote work performance,^[146]^ and work-family policies that promote happiness and employee commitment.^[147]^ That underscores the importance of PLS-SEM in studying the variables related to WFB.
3. The earliest co-cited types of literature were LT Eby, 2005 (19 citations, centrality 0.04), K Byron, 2005 (17 citations, centrality 0.01), and JH Greenhaus, 2006 (26 citations, centrality 0.08). These references span from 2007 to 2010 and are widely recognized. Specifically, in the study, LT Eby reviewed research on work-family relationships from 1980 to 2002. He addressed key topics, such as WFC, gender linkage, role stress, dual-working parents, assistance programs, and time arrangements. Additionally, he identified the following research gaps: How do children affect WFC? Are there personal expectations for work and family fulfillment?^[148]^ Additionally, Greenhaus proposed that work-family enrichment (WFE) can be achieved through instrumental pathways (effective communication) and affective pathways (positive emotional spillover).^[149]^
4. The most recent co-citations were by V. Braun, 2021 (39 times, 0.04); L Craig, 2021 (14 times, 0.03); and H Chung, 2020 (14 times, 0.02). Notably, V Braun has the latest co-citation year (2021) and the highest count of co-citations (39), which is recognized for its significant contributions to topic analysis methods. Additionally, Chung explored how flexible work impacts gender roles and WFB, finding that women often take on more family responsibilities, while men pursue professional growth. He advocates breaking traditional gender roles to promote equal attention to work and family for both genders. He also suggests future research directions: the long-term effects of flexible working on the gender compensation gap, and a comparative analysis of flexible working systems across countries.^[39]^

In conclusion, some highly cited references discuss research methods, such as subject analysis and PLS-SEM, while others summarize past studies and suggest future directions. Additionally, some studies have highlighted its practical significance. This indicates that future researchers should provide valuable references for their work.

**Figure 9.**
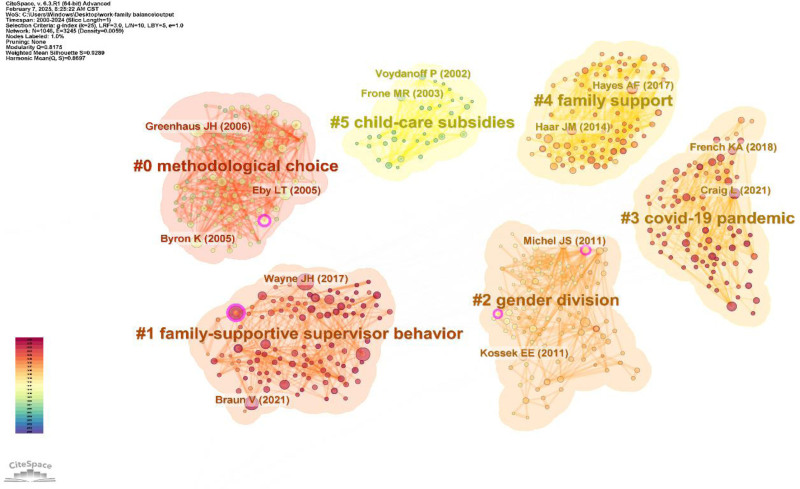
Cluster graph of references co-citation (node = 1046; link = 3245).

Figure 9 presents the reference co-citation cluster graph with 1046 nodes and 3245 links. The analysis was performed as follows:

1. Some clustering methods focus on WFB, such as #0 (methodological choice), which includes questionnaire surveys, semi-structured interviews, meta-analyses, and literature reviews. Byron K’s notable meta-analysis examined 2 types of WFC: work interfering with family and family interfering with work. His study found that women are more affected by work interfering with family, whereas men tend to experience family interfering with work due to family pressure, conflict, number of children, and marital status.^[150]^ This method is widely used in the WFB research.
2. Some clusters emphasize the importance of organizational, family, and social support factors in achieving WFB. Key clusters included #1 (family supportive supervisor behavior), #4 (family support), and #5 (childcare subsidies). Cluster #1 emphasizes that enterprises can boost employee performance by encouraging family oriented managerial behaviors and fostering internal status and emotional commitment.^[151,152]^ Key references in this cluster include JH Wayne’s research, which demonstrates that supportive supervisors can alleviate the negative impact of work on family life and improve employee satisfaction.^[55]^ Cluster #4 highlighted the significance of family support in maintaining WFB. This support, which includes resource provision, emotional backing, and practical assistance, enhances the work-family interface and mitigates resource depletion at work, consistent with COR theory. The representative co-cited references in this cluster suggest that family networks are particularly beneficial in collectivist cultures by helping individuals manage anxiety and depression while reducing reliance on WFB.^[153]^ Cluster #5 focuses on childcare subsidy policies to reduce WFC. Voydanoff proposed strategies that use childcare subsidies to alleviate these conflicts.^[154]^
3. Clusters also examined individual and environmental factors affecting WFB, such as #2 (gender division) and #3 (COVID-19 pandemic). Cluster #2 (gender division) highlights the differences between men and women in housework, childcare, and labor. Combined with #3 (COVID-19 pandemic), this shows that mothers typically bear a heavier burden of family responsibilities during the pandemic. However, when fathers actively engage in household chores and childcare, their relationship satisfaction is enhanced.^[155]^ Among them, a key reference for cluster #2 is a 2010 article by JS Michel, which explores how individual gender and factors such as marital and parenting status influence WFB. Married men with children often face fierce workplace competition due to family pressures, disrupting their WFB. By contrast, married women typically limit their career development because of their family responsibilities.^[156,157]^ Cluster #3 examines the impact of the COVID-19 pandemic on WFB. A significant study by Craig investigated paid work and unpaid family care for working parents. The findings reveal that while fathers became more involved in childcare during the pandemic, mothers took on more unpaid labor overall and reported lower satisfaction with their work-family relationship; fathers also expressed greater dissatisfaction compared to pre-pandemic levels.^[158]^

In summary, WFB studies commonly focus on research methods, support factors, and individual influences. We encourage future researchers to explore new perspectives to gain practical insights.

#### 3.3.4. Summary

In conclusion, co-citation analysis of the WFB has established the foundation of this field. The identified clustering themes remain a key topic for future research. For example, a timeline analysis of author co-citations suggests that future studies should focus on WFC, time use, job satisfaction, gender issues, and mothers in the workplace. Cluster analysis of journal co-citations shows that factors such as WFB enhancement, gender role differences and equality, job satisfaction, and family satisfaction remain crucial in the WFB domain. Additionally, reference co-citation highlights that research methods, support factors, and individual influences retain relevance.

### 3.4. Co-occurrence characteristics analysis

Co-occurrence analysis examines the relationships between categories and keywords in the text, revealing research trends and future directions. This section identifies the key disciplines in WFB research, keyword evolution, and emerging hot topics. This addresses the fourth question: What are the emerging hotspots and key trends in this field?

#### 3.4.1. Category co-occurrence

Table 10 lists the top 10 co-occurrence categories, showing their frequency, centrality, and starting time. These categories encompass management and public health, family society, psychology, and economic and social sciences, highlighting key themes in the field.

**Table 10:** Top 10 category co-occurrence in WFB field.

Ranking	Category	Count	Centrality	Year
1	Management	196	0.14	2000
2	Public, Environmental & Occupational Health	172	0.33	2000
3	Applied Psychology	150	0.11	2000
4	Family Studies	144	0.00	2001
5	Sociology	110	0.59	2001
6	Multidisciplinary Psychology	94	0.00	2000
7	Women’s Studies	70	0.34	2002
8	Interdisciplinary Social Sciences	69	0.15	2000
9	Economics	60	0.10	2004
10	Industrial & Labor Relations	59	0.14	2001

WFB = work-family balance.

1. Management and public health often intersect within areas such as management (196), public, environmental, and occupational health (172), and industrial relations and labor (59). This suggests that research not only focuses on occupational health but also examines the dynamics of industrial relations. For example, studies indicate that enhancing women’s employment rates in the private sector requires organizational support and family friendly policies beyond current family assistance to promote harmonious industrial relations.^[159]^
2. The family social category has a high centrality. Sociology showed the highest centrality (0.59), indicating strong ties with co-occurrence networks and emphasizing the importance of WFB in social phenomena. Women’s studies also demonstrated a significant centrality at 0.34, reflecting their impact on the field and leadership in co-occurrence networks. Neoliberal feminists argue that, as WFB becomes more prominent and women seek to be working mothers instead of stay-at-home mothers, we should advocate eliminating traditional gender differences in the workplace.^[160,161]^
3. Psychological disciplines, such as applied psychology (150 times, 2000) and multidisciplinary psychology (94 times, 2000), enhance the study of WFB and support individuals’ mental health. Research based on self-determination theory (SDT) shows that need satisfaction, adequate sleep, and job autonomy improve the mental health of working mothers,^[162]^ highlighting the theory’s focus on the need for autonomy.
4. Social and economic disciplines demonstrate strong performance, notably interdisciplinary social sciences (69 times total, 0.15, 2000), and economics (60 times total, 0.10, 2004). This suggests that social science theories and economic analyses are pertinent to WFB studies. For example, some research has applied COR theory to create a model connecting decent work, WFC, fertility intention, and social support.^[163]^ The findings show that social support moderates the link between decent work and WFC, while decent work boosts fertility intentions by alleviating WFC.^[164]^

In short, these co-occurrence categories encompass core research on the WFB, highlighting its interdisciplinary nature.

**Figure 10.**
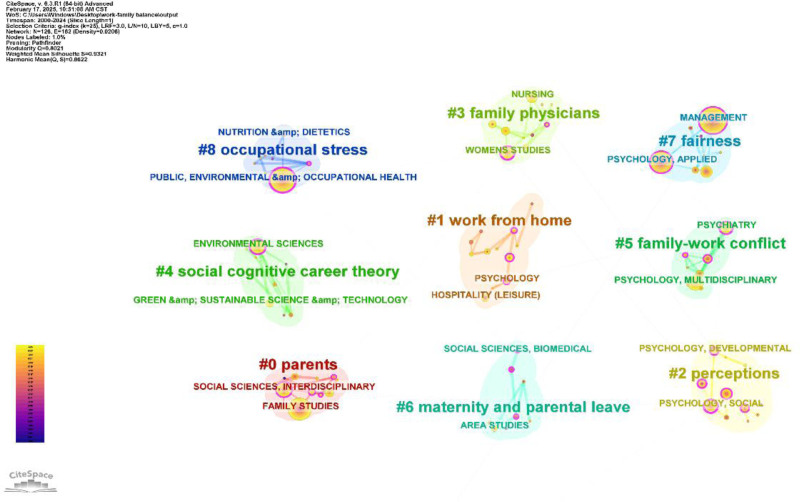
Cluster graph of category co-occurrence (node = 126, link = 294).

Figure 10 shows the co-occurrence cluster diagram, emphasizing the research subfields of the WFB. The analysis was performed as follows:

1. Some clusters focused on family factors, such as #0 (parents), #1 (work from home), and #5 (family-work conflict). Cluster #0 (parents) integrates boundary theory and role theory to examine WFB topics, including parents’ well-being and parenting quality in childcare, vacation, flexible employment, and emotional health. Meanwhile, #5 (family-work conflict) shows that WFC can lead to parental burnout, negatively impacting children’s education. This issue can be alleviated by parents’ optimistic mindset.^[165]^ Cluster #1 (work from home), represented by psychology, examined the willingness to work remotely. Research shows that emotional support from spouses and job-related assistance enhance the desire to work from home. Furthermore, sharing household duties and childcare improves WFB.^[166]^
2. Some clusters focused on occupational factors, including #3 family physicians (exploring the WFB of the profession of family doctor), #6 maternity and parental leave (leave for working mothers), #7 fairness (equitable treatment by management), and #8 occupational stress (stress levels across occupations). Notably, cluster #3 emphasizes the link between work stress, burnout, and WFB among family physicians, with the representative co-occurrence categories being nursing and women’s studies. Research indicates that work stress faced by family physicians can lead to burnout and decreased engagement; thus, improving WFB may help reduce their stress levels.^[66]^ Cluster #7 explores factors affecting perceptions of unfairness: high effort with low rewards, diminished WFB,^[167]^ and advocating for fair management of working mothers.^[168]^
3. Other clusters focused on research perspectives and theoretical applications, such as #2 perceptions (highlighting diverse viewpoints) and #4 social cognitive career theory (supporting career choices by examining human behavior and decision-making through a triadic model of personal, environmental, and behavioral factors). Cluster #2 includes representative categories such as social psychology and developmental psychology. This study explores family labor division, supportive incentives, and fathers’ involvement in child-rearing within WFB through the lenses of COVID-19, supervisors, and gender roles.^[169–171]^ Cluster #4’s representative categories were environmental sciences and green and sustainable science and technology. Social cognitive career theory integrates individual factors with the environment and behavior. The findings show that a positive work environment enhances job retention, while WFB promotes career development.^[172]^

In summary, the co-occurrence categories revealed diverse subfields, with family, profession, perspective, and theory as key academic themes. They also illustrate the interdisciplinary nature of management, public health, psychology, family studies, social sciences, and economics.

#### 3.4.2. Keyword co-occurrence

Keyword co-occurrence refers to the simultaneous appearance of 2 or more keywords in a single article. Table 11 shows the co-occurrence frequency, centrality, and initial occurrence time of keywords related to WFB. The analysis was performed as follows:

**Table 11:** Top 10 keyword co-occurrence in WFB field.

Ranking	Keyword	Count	Centrality	Year
1	conflict	290	0.03	2000
2	work-family balance	280	0.04	2003
3	gender	210	0.08	2001
4	work-family conflict	175	0.03	2001
5	satisfaction	161	0.04	2000
6	impact	154	0.07	2000
7	stress	141	0.06	2001
8	job satisfaction	140	0.04	2007
9	health	138	0.04	2004
10	women	137	0.05	2000

WFB = work-family balance.

1. The top 3 keywords were “conflict” (290 times), “work-family balance” (280 times), and “gender” (210 times). Among these, “work-family balance” is the central theme. A recent study found that racial microaggressions and traditional gender stereotypes faced by working mothers of color hindered their WFB, leading to burnout. It highlighted the importance of supportive work environments in reducing burnout,^[173]^ suggesting that WFB research should address cultural background, racial differences, gender disparities, and psychological factors. Additionally, these co-occurring keywords emphasized the goals of WFB research: alleviating conflict, enhancing WFB, and promoting gender equality.
2. The top 3 centrality keywords are “gender” (0.08), “impact” (0.07), and “stress” (0.06), reflecting a wide range of topics addressed. Notably, “gender” is the most central keyword and the third most common, highlighting the WFB’s emphasis on gender differences, equality, roles, and the application of gender role theory. Research indicates that traditional gender roles often lead women to take on more family responsibilities, heightening their risk of WFC due to the imbalances stemming from these norms. This suggests that achieving WFB and gender equality requires equal sharing of work and family duties among spouses.^[174]^ Furthermore, “impact,” which has high centrality and an early co-occurrence time, highlights WFB’s enduring influence in studies on effects on individuals, families, organizations, and societies. The high centrality of “stress” highlights its extensive thematic connections, serving not only as a core challenge for WFB, but also as a key mediator and predictor of WFB, along with variations in health, satisfaction, performance, and gender.
3. The earliest co-occurring keywords were “conflict,” “satisfaction,” “impact,” and “women,” all emerging in 2000. They underscore the dual conflict that female employees face as mothers, affecting WFB and decreasing job-family satisfaction, longstanding research concerns. Recent studies indicate that excessive work-life stress reduces women’s time use satisfaction, resulting in prolonged negative emotions. Furthermore, childcare services may not effectively address this issue as they can increase financial pressure on female workers, exacerbating conflicts that harm mental health and WFB.^[175]^
4. The keywords “job satisfaction” (2007) and “health” (2004) emerged later, indicating that their significance was initially overlooked. As research has evolved, these topics have become central concerns. Studies have shown that unstable work significantly contributes to childcare stress among Mexican immigrant women. They often work overtime and endure exploitation to keep their jobs, leading to low job satisfaction, which adversely affects their mental health and WFB. Addressing this issue necessitates anti-exploitative immigration reform and national labor legislation.^[176]^

In summary, high-co-occurrence keywords are central to WFB research, reflecting their integrated content. Studies address gender differences, WFB, mental health, and stress, whereas gender difference studies focus on women, conflict, satisfaction, and influencing factors. This underscores the diversity within the WFB research.

**Figure 11.**
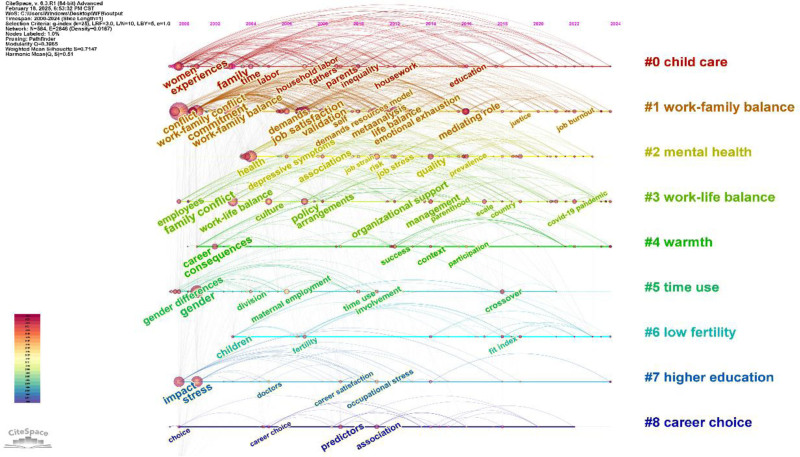
Timeline of co-occurrence keywords (node = 584; link = 2846).

Figure 11 shows the timeline of keyword co-occurrence, highlighting 2 key aspects: First, related keywords are clustered, reflecting the evolution of the research field. Second, these co-occurring keywords reveal the core focus and research hotspots in the literature.

1. The clusters with the longest co-occurrence times are #0 (childcare), #1 (work-family balance), #3 (work-life balance), and #7 (higher education). Notably, #1 and #3 recently emerged as prominent keywords. In the early stage of cluster #0 (child care) from 2000 to 2006, common keywords included “women,” “experiences,” “family,” “time,” and “labor,” focusing on working mothers’ career experiences. Research has indicated that family factors, such as having young children or a non-employed spouse, affected women’s WFB more than men did.^[177]^ From 2007 to 2024, key terms shifted to “household labor” and “inequality.” Studies have interviewed scholars and working mothers about their experiences with WFB inequality and household responsibilities. The findings revealed that the pandemic reduced work flexibility for mothers while increasing their housework and childcare duties.^[178]^ Cluster #1 (work-family balance) has focused on the conflict factors influencing WFB since 2000, and introduced the demands-resources model in 2010. A meta-analysis was started in 2011 to assess WFB outcomes by exploring the balance between job demands and available resources. Recently, #1 investigated the negative impacts of social media addiction, which contributes to emotional exhaustion, increased job burnout, challenges in achieving WFB, and reduced job performance.^[179]^ Decent work mitigates negative outcomes and boosts employee engagement and employability. Leader involvement fosters innovation and reduces job burnout.^[180]^ Cluster #3 (work-life balance) identifies WFB as a key research area, emphasizing health and family time scheduling.^[181]^ Key terms include “employees,” “family conflict,” and “organizational support,” with the latter being crucial for regulating WFB. A survey of medical staff in Guangdong, China, found that support from health administration and relevant government departments improved WFB and job satisfaction among healthcare workers.^[182]^ The emergence of COVID-19 in 2021 further increased the scholarly focus on WFB related to this public health crisis. Cluster #7 (higher education) highlights “stress,” “career satisfaction,” and “occupational stress” as key co-occurring keywords in this subfield within the WFB. A recent study examined work-family imbalance among middle-aged female higher education managers, revealing that heavy workloads and social career expectations contribute to work stress.^[183]^
2. The cluster with the shortest co-occurrence time was #2 (mental health), which covers 2004 to 2023. Key co-occurring keywords include “health,” “depressive symptoms,” “associations,” and “quality,” highlighting a focus on mental health, predominantly depressive symptoms. Factors influencing mental health, such as quality of family relationships, were also examined.^[184]^ Here, “health” refers to college students’ mental wellbeing. Research indicates that 22.2% of college students experience moderate depression; low WFB correlates with increased anxiety and stress, whereas happiness can mitigate these negative emotions.^[185]^
3. The latest cluster is cluster #6 (low fertility), covering 2003 to 2024. Research on “fertility” indicates that while many young people wish to become parents, they are increasingly aware of the professional and familial challenges women face in childbearing. Couples who are career-negative, financially underemployed, or infertile often have more positive relationships, suggesting that they may delay or forgo having children because of WFB concerns.^[184]^

In summary, this underscores the WFB’s long-term research focus and keyword evolution, enhancing readers’ understanding of the field.

#### 3.4.3. Keyword bursts

**Figure 12.**
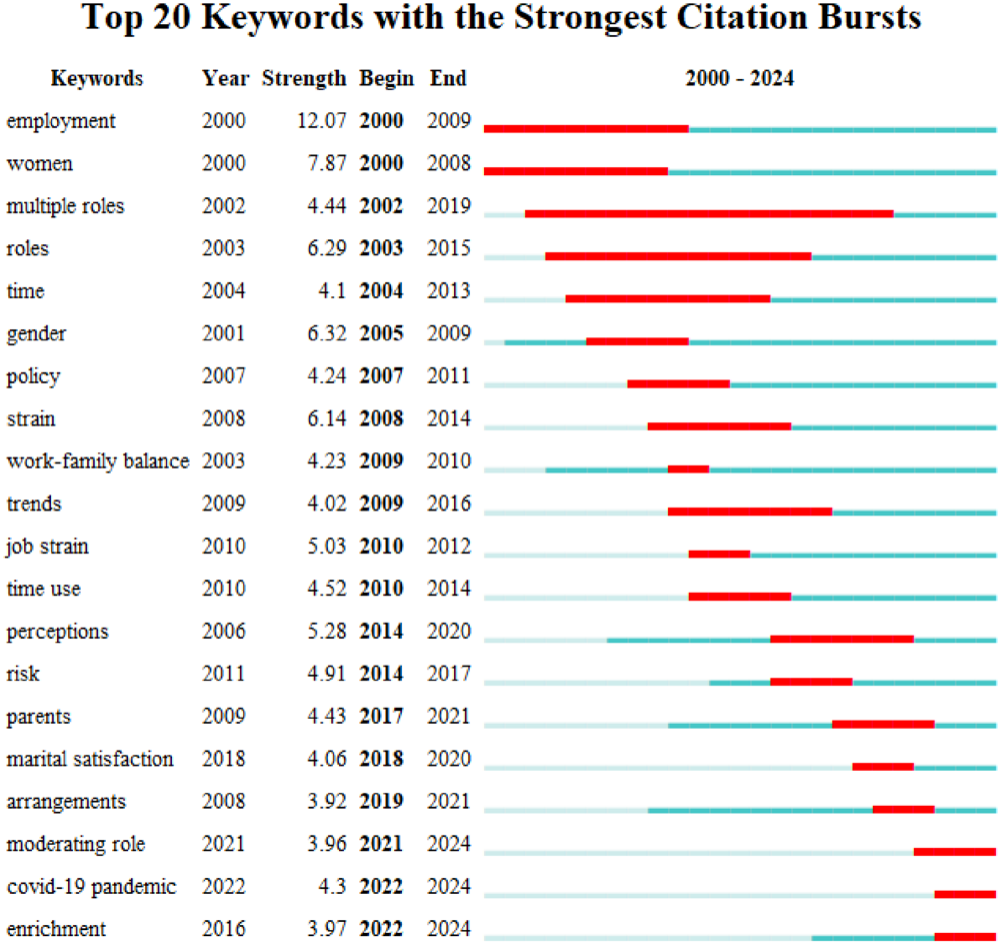
Burst analysis of top 20 keywords.

A keyword citation burst refers to a sudden spike in keyword citations in WFB research articles over a specific period. Figure 12 displays the top 20 burst keywords for WFB, with the red line indicating the outbreak duration and the dark blue line representing the keyword duration. Details are as follows:

1. Key terms that emerged include “employment” (2000), “women” (2000), “multiple roles” (2002), “roles” (2003), “time” (2004), and again “employment” (2000). This underscores the focus on working women’s multiple roles, such as childcare, housework, caregiving, and time management in families, during early WFB research, laying a foundation for the field. This demonstrates how gender role theory creates specific challenges for women in the workplace.
2. The keywords with the highest burst strength were “employment” (12.07), “women” (7.87), “gender” (6.32), “roles” (6.29), and “strain” (6.14). Notably, “employment” and “women” had the highest strength and earliest emergence (from 2000), highlighting that women’s balance between work and family, as well as their coping with pressure, are central issues in WFB. Additionally, gender role differences, such as women typically bearing more family responsibilities, which can hinder their career development, and stress within working families are significant topics in WFB research. These high-intensity keywords underscored the impact of women, roles, and stress in this field.^[186]^
3. The keywords with the most extended burst duration were “multiple roles” (17 years) and “roles” (12 years), each showing different hotspots at various research stages, reflecting the evolution of role theory and gender role theory. From 2000 to 2010, articles citing “multiple roles” addressed conflicts arising from diverse family structures and proposed strategies that integrate family friendly policies with work-family culture to alleviate these issues.^[187]^ From 2010 to 2024, this research highlighted the benefits of multiple roles, such as enhanced personal resources and reduced depressive symptoms.^[188]^ Additionally, topics like external role support and role balance were discussed about “multiple roles.”^[189]^ From 2000 to 2010, studies on “roles” emphasized positive factors influencing WFB, including fairness and low job autonomy, rather than merely role stress and differences.^[190]^ Between 2010 and 2024, a significant topic emerged regarding gender role differences in marriage and childbearing intentions. The findings revealed substantial disparities between men’s and women’s views; couples with egalitarian perspectives tended to have lower fertility intentions. Societies can enhance fertility through supportive WFB measures.^[191]^
4. Recent keywords highlight key topics in the WFB. In 2018, “marital satisfaction” became a significant issue linking marital dynamics to WFB. Research has shown that economic and parenting pressures have a negative impact on marital satisfaction.^[192]^ By 2019, the focus had shifted to working family arrangements, emphasizing flexible work options, prioritizing urgent tasks, dividing family duties, and sharing responsibilities equally.^[174]^ The “moderating role” concept gained prominence in 2021 as researchers identified factors enhancing WFB. Positive influences include organizational support, family support, available resources at home and work, and a supportive work-family culture.^[193]^ The “COVID-19 pandemic” sparked discussions on remote work, gender inequality in roles, and family well-being within the WFB framework in 2022.^[194]^ “Enrichment” has been increasing since 2022 and continues today. The positive spillover of WFE on the family-work interface is a key aspect of the WFB approach. For example, some studies examine how leadership improves WFE for managers using COR theory.^[195]^ Some studies have found that WFE mediates the relationship between family boundary flexibility and work engagement based on person-environment fit theory. This promotes WFE through family support, leading to improved work engagement performance.^[5]^ Some scholars have examined the effects of 2 types of work passion (harmonious and obsessive) on prosocial behavior in employees and their spouses using JD-R theory and crossover theory. They found that harmonious work passion enhanced WFE, leading to increased prosocial behavior. In contrast, obsessive work passion heightens WFC and reduces prosocial behavior in both employees and their spouses.^[65]^ A study based on JD-R theory found that higher work demands during nonworking hours reduce women’s WFE. Emotional imbalance negatively affected WFE in both genders. Increased supervisory coaching improves WFE.^[194]^ Additionally, role theory and boundary theory can also be used to discuss the positive impact of spousal support on WFE.

In summary, keywords related to the WFB reflect current research trends and their evolution. Key topics included marital satisfaction, work-family arrangements, positive factors enhancing WFB, the impact of COVID-19, and WFE. These themes are prominent, and will remain relevant in the near future.

#### 3.4.4. Summary

Co-occurrence analysis highlights category co-occurrence, evolution of hot keyword co-occurrence, and keyword bursts. It was found that management and public health have a high co-occurrence frequency, specifically in areas such as Management, Public, Environmental & Occupational Health, and Industrial Relations & Labor. Future research should focus on how organizations can foster harmonious industrial relations through flexible working hours, remote work options, parental leave, and supervisor support to identify new measures that enhance WFB. In addition, psychology and family sociology are foci of co-occurrence. In the future, healthy family relationships and WFB in different family structures will be explored. Scholars also need to adopt an interdisciplinary perspective to comprehensively examine WFB. Keywords with higher co-occurrence frequencies included conflict, WFB, gender, and others. The key clustering themes included childcare, WFB, work-life balance, mental health, and low fertility rates. It is important to note that low fertility rates are a current research hotspot owing to declining birth rates in several countries. The emergence of keywords suggests that “moderating role,” “COVID-19 pandemic,” and “enrichment” remain prominent topics. Notably, “enrichment” will continue to be integrated with mainstream theories to explore how to achieve WFE and its associated benefits.

## 4. Theoretical insights

### 4.1. Domain knowledge framework

**Figure 13.**
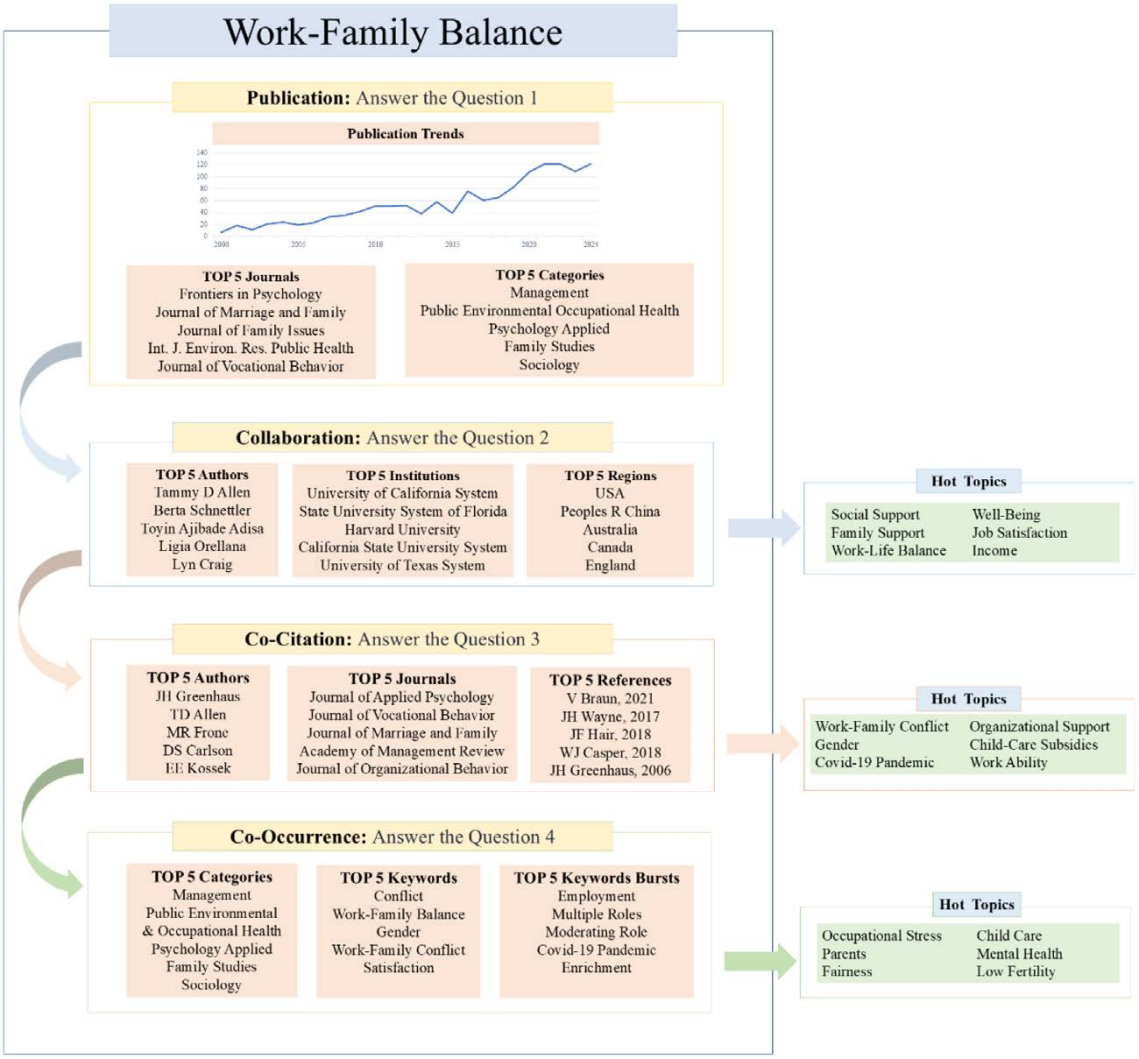
Knowledge framework of work-family balance.

The WFB research field is extensive and fragmented, encompassing various subfields that complicate a comprehensive understanding of the existing findings. Therefore, a systematic review of WFB research is crucial for clarifying its foundation, relationship, status, and prospects. Figure 13 illustrates the WFB panoramic knowledge framework based on 4 analyses: statistical, collaboration, co-citation, and co-occurrence. This framework provided an overview of the WFB research landscape. The statistical analysis section presents the annual publication volume of WFB-related research, including journals and subject categories, it answers Q1: Has this research area gained significant attention? The collaboration analysis section visualizes active authors, institutions, and regions through clustering diagrams, highlighting key relationships and themes to address Q2: What are the key relationships and collaboration themes in this field? The co-citation section analyzes key co-cited authors, journals, and references using timeline graphs and clustering diagrams to answer Q3: What is the status of influential research in this field? The co-occurrence section examines keywords and subject category themes using timeline graphs to illustrate the keyword co-occurrence. It also produced a keyword burst evolution graph, collectively answering Q4: What are the emerging hotspots and key trends in this field?

1. To address question 1, statistical analysis was the foundational section of the WFB. This section highlights the popularity of WFB’s theme research, key publishing journals, and advantageous disciplines by analyzing annual publication volume, journal types, and discipline categories, providing practical insights for scholars. The results showed a steady increase in WFB publications on WFB. “Frontiers in Psychology,” “Journal of Marriage and Family,” “Journal of Family Issues,” “International Journal of Environmental Research and Public Health,” and “Journal of Vocational Behavior” are most receptive to submissions in this field. Management, Public Environmental Occupational Health, and Applied Psychology researchers have demonstrated significant interest in this area.
2. To answer question 2, collaboration analysis shows the connections among the authors, institutions, and countries of the WFB. This section offers insights into potential collaborators and themes. Active authors include Tammy D Allen, Berta Schnettler, and Toyin Ajibade Adisa. Leading institutions with strong collaboration intentions are the University of California System, the State University System of Florida, and Harvard University. The top 5 countries showing high collaboration enthusiasm were the United States, China, Australia, Canada, and the United Kingdom. Prominent collaborative topics include support for WFB (social support and family support), employees’ happiness at work (well-being and job satisfaction), broader work-life balance issues beyond WFB, and family economic concerns (income).
3. Co-citation analysis addresses question 3 by highlighting highly co-cited authors, journals, and references. This analysis helps readers to understand the current knowledge landscape. The top 5 co-cited authors were JH Greenhaus, TD Allen, MR Frone, DS Carlson, and EE Kossek. Notable journals with high co-citation counts include “The Journal of Applied Psychology” and *The Journal of Vocational Behavior*. Frequently cited references include Braun (2021), Wayne (2017), and Hair (2018). Key clustering topics included individual differences (gender, work ability), means to support WFB (organizational support and childcare subsidies), and environmental factors influencing WFB (COVID-19 pandemic, WFC).
4. Co-occurrence analysis is the prospect section of the WFB, addressing question 4 by highlighting evolving trends and dynamic changes in keyword hotspots. This includes category co-occurrence, keyword co-occurrence, and keyword bursts. “Management,” “Public Environmental Occupational Health,” etc, appear in co-occurrence and statistical category analysis. The top 5 frequently mentioned keywords were “conflict,” “work-family balance,” “gender,” “work-family conflict,” and “satisfaction.” Notably, “employment” shows the highest burst intensity; “multiple roles” has the most extended burst duration; “moderating role,” “covid-19 pandemic,” and “enrichment” are recent hot keywords. These popular categories and keywords highlight key topics: social phenomena related to WFB (low fertility), personal health (mental health), parental roles (parents, childcare), workplace stress, and fairness issues. These themes will continue to attract scholarly attention.

### 4.2. Future research

Based on a bibliometric analysis of the past 24 years, we have identified trends, key topics, and evolving hotspots in WFB, establishing a framework for a comprehensive study. This section further explores future development trends and research characteristics of WFB by examining its key subfields and hot keywords.

#### 4.2.1. Future research should adopt an interdisciplinary approach

Articles on management and public health will remain relevant in the future. According to the category statistics and category co-occurrence analysis from previous studies (Tables 3 and 10), Management and Public Health disciplines show high co-occurrence frequencies in the WFB field. Specifically, Management had a frequency of 196, Public, Environmental & Occupational Health had a frequency of 172, and Industrial Relations & Labor had a frequency of 72. Cluster #7 (fairness) in Figure 10, where management is located, examines how management ensures the fair treatment of employees to support their WFB and enhance organizational contributions. Cluster #8 (occupational stress) in Figure 10, associated with Public, Environmental & Occupational Health, investigates the pressure to balance demands across various occupations. Their themes may explore how organizations can foster harmonious labor relations through flexible working hours, remote office locations, parental leave, and supervisory support. This includes the examination of new measures that promote WFB and transform occupational stress into motivation.

The co-occurrence of the disciplines of psychology, namely Applied Psychology (with a co-occurrence frequency of 150 times) and Multidisciplinary Psychology (94 times), began in 2000 and continues today, highlighting its fundamental role in WFB. The co-occurrence tags of the psychological discipline as shown in cluster #1 (work from home) in Figure 10 explore the connection between the willingness of couples to work from home and WFB, while cluster #5 (family-work conflict) studies the psychological mechanisms behind the conflict. In the future, the effects of work burnout, depressive mood, satisfaction, happiness, and positive affect spillover on WFB will be explored.

Family social disciplines, including Family Studies, Sociology, and Women’s studies, exert a significant influence, with sociology having the highest centrality (0.59). Cluster #0 (parents) in Figure 10 encompasses Family Studies and focuses on WFB related to parental well-being and quality. Future research will investigate healthy family relationships and WFB across diverse family structures, such as the impact of single-parent families on parents, children, and grandparents, as well as compare WFE between separated/divorced families and traditional families. The role of multigenerational family resources in WFB is also examined.

Moreover, future research should focus on interdisciplinary studies. For example, Sociology and Interdisciplinary Social Sciences will be combined to examine how social phenomena, such as higher education, low fertility rates, population aging, flexible work, technology’s integration of work into family life, and robot nannies, affect WFB and the corresponding strategies.

Ultimately, future research should integrate findings from various scientific fields to provide a holistic perspective. This approach highlights the diverse contributions of experts across disciplines, thereby fostering comprehensive development in the field.^[196]^ For instance, while management scholars, psychologists, and sociologists focus on WFB, geographers can examine how home-workplace boundaries affect urban resource distribution. Design experts might look for ways to minimize urban commuting distances, and legal scholars may analyze the interplay between labor laws, such as paid parental leave and flexible working hours, and compliance costs for businesses.

#### 4.2.2. Future research should explore more complex themes

Our analysis indicated that WFB encompasses various topics, including WFC, satisfaction, role differences, support mechanisms, working mothers, gender issues, mental health, and economic pressure. This underscores the extensive scope of WFB research in addressing current conflicts, while examining psychological mechanisms and gender disparities. Future studies should investigate the division of household labor, parenting roles, family status, and career advancement between men and women and their effects on WFB satisfaction.

Existing decent work scales address job security, support, self-esteem, self-value, and well-being.^[197,198]^ In the future, a more authoritative WFB scale will be developed to measure balance. Moreover, the latest emerging keywords such as “moderating role” (strength 3.96), “covid-19 pandemic” (strength 4.3), and “enrichment” (strength 3.97) have all emerged since 2021 and continue to be relevant through the end of 2024. This suggests that these keywords represent new hotspots in WFB research compared to others. In the short term, researchers will focus on topics such as the “moderating role” of positive factors that influence WFB. In the future, they may investigate how organizational, family, and individual factors, such as parental leave policies, leadership styles, flexible employment options, family resources, and self-emotional regulation, affect employees’ WFB.

The most challenging year of the COVID-19 pandemic has passed since then. However, its impact on physical and mental health, remote work, household chores, economic pressure, and parenting has sparked extensive discussion among scholars about WFB (as shown in cluster #3 COVID-19 in Fig. 9). In the future, discussions will focus on changes in parenting models post-pandemic, gender division of labor, and the redefinition of work-family boundaries in hybrid office settings. The COVID-19 pandemic is a significant part of the historical context of the WFB knowledge domain.

Moreover, beyond COVID-19, other sudden public events may spark global discussions on WFB related to economy, health, humanism, and technology. For instance, as the Internet rapidly evolves alongside AI in the digital age, exploring how to establish a new WFB paradigm in this context is crucial.

“Enrichment” refers to the positive gain process of an individual’s work and family experiences. The co-cited reference JH Greenhaus (2006) in Table 9 indicates that enrichment arises from positive spillovers and has garnered significant attention (26 co-citations, centrality of 0.08). Future research should focus on achieving positive interactions between work and families to enrich both. This may include methods for WFE, the resulting positive psychological changes, and how such psychology promotes WFB. Moreover, new research theories have continued to emerge. For example, COR theory will be used to examine how resource replenishment supports WFE, while JD-R theory focuses on the interaction between job demands and resources. In the future, we may also investigate how job demands hinder WFE development. The person-environment fit theory highlights the importance of aligning the work environment with employee capabilities and values to enhance WFE. Role theory examines how individuals leverage resources across multiple roles in order to create a reinforcing cycle. The spillover model supports the positive impact of emotional and skill transfer at the work-family interface. Boundary theory emphasizes how individuals manage the permeability and segmentation of work-family boundaries to minimize WFC. In conclusion, research on enrichment will focus on resource accumulation and demand management to explore the positive spillover and transformation between work and family, ultimately enhancing happiness.

#### 4.2.3. Future research should explore various theories and methods

At the theoretical level, the JD-R model is central to the WFB research. It explores how work resources, such as organizational support, commitment, work environment, culture, career development opportunities, and family supportive leadership, interact with work demands, such as working hours, pressure, and emotional exhaustion. Future studies should integrate new working models to further investigate the impact of these factors on WFB. (Table 7 highlights co-cited author Bakker’s key contributions related to JD-R; Fig. 7’s #4 on burnout illustrates emotional depletion due to work demands, emphasizing JD-R’s relevance.) COR theory is applied to research on preserving resources for WFB in a cross-cultural context. The highly cited work of co-cited author SE Hobfoll, as shown in Table 7, introduces COR theory. Figure 6 (#5 family support) explores how family resources create a gain cycle for WFB, while Figure 9 (#4 family support) discusses how the family’s systematic support network compensates for workplace losses, emphasizing the importance of the COR theory. Boundary theory will be used to examine how remote work and digital technology affect work-family boundaries. SDT explores the role of basic psychological needs in achieving WFB (the analysis in Table 10 of Psychological Disciplines highlights the significance of the autonomy need in SDT). Gender role theory will be utilized in the future to examine the link between flexible gender roles, such as men taking on more family responsibilities, women pursuing careers, and subjective well-being (as illustrated in Fig. 6 #1 subjective well-being, Fig. 7 #3 gender, Fig. 8 #1 gender roles, and #7 gender equality). Additionally, it will explore policy design for a high-welfare society and analyze various marginalized groups, including the unique challenges faced by immigrant women and family physicians in balancing work and family (as shown in Fig. 6, #6 family physicians).

In addition, theories from other disciplines can be applied to WFB research. However, new theories continue to emerge. For example, positioning theory may help interpret how gender norms shape the roles of “ideal worker” and “caregiver.” The technology acceptance model, in the context of AI and remote work, can examine how technology affects the work-family interface. Life-history theory offers an evolutionary biological perspective on low fertility rates, focusing on fertility, higher education, and working mothers in WFB (see #6 working mothers in Fig. 7; #6 low fertility and #7 higher education in Fig. 11). In the future, it may integrate with sociology to explain how educated women balance fertility and career growth through resource allocation (time, energy, finances, and educational investment).

At the analytical method level, future studies will combine qualitative methods (e.g., interviews, focus groups, case studies) with quantitative approaches (including meta-analysis, PLS-SEM, and path analysis) and integrate methodologies from other fields (e.g., fsQCA, coupling coordination, and gray prediction).

#### 4.2.4. Future research should emphasize cross-regional and cross-cultural cooperation, and WFB comparisons

As more countries engage in WFB studies, international exchange has become crucial. Future research will focus on the comparative performance of WFB across different cultural backgrounds in various countries, the impact of cultural differences on employees’ WFB in multinational enterprises, and practical implications for nations with high WFB satisfaction, such as Canada and the Netherlands. Moreover, in cross-regional trade, the WFB can act as a threshold or tool for facilitating trade. We also discuss the mechanisms and effects of these policies on different trade entities. Furthermore, countries with low WFB satisfaction often face challenges in balancing work-life balance, economic development, and carbon neutrality. This exploration examines how these countries can achieve sustainable WFB development and the lessons their practices offer to others. The United States will remain the leading region in this field with 489 collaborations (see Table 6). The United Kingdom, Germany, and Australia will continue to lead the collaboration network with centrality values of 0.52, 0.29, and 0.12, respectively (see Table 6). Some developing countries actively seek partnerships; for example, China must collaborate more widely to enhance its influence. Although China ranked second in collaboration numbers (as shown in Table 6), its centrality was only 0.04.

## 5. Discussion

### 5.1. Key findings

This study uses CiteSpace to conduct a bibliometric analysis of 1390 articles on WFB from the core database, providing an overview of WFB research. The findings include statistical, cooperative, co-citation, and co-occurrence analyses, which address 4 key questions.

1. Statistical foundation: Recently, particularly after the emergence of COVID-19, discussions on WFB have increased significantly. Annual publication volumes are sharply rising in various psychology and family studies journals, as well as in work and economic relations journals, underscoring the interdisciplinary nature of management, psychology, social sciences, and economics.
2. Key collaborative connections and themes in this field: The WFB author collaboration group operates independently, with strong internal ties. Teams led by Ligia Orellana and Berta Schnettler, which have more members, focused on family satisfaction and WFB. Research cooperation between European and American institutions is well established, with the University of London as a key player. Institutional cooperation themes included gender income gaps, job satisfaction enhancement, and support for family friendly workplaces. Countries with strong human rights protections (e.g., Canada, the Netherlands, and Germany) more frequently cooperate in social support, individuals, and psychological well-being.
3. Current status of influential figures in this field: Co-cited authors like JH Greenhaus emphasize work-family role overlap, defining “balance” as a psychological sense of satisfaction. In contrast, SE Hobfoll developed influential theories in WFB research, such as COR. AB Bakker promoted the use of JD-R to analyze WFC to achieve WFB. Key co-cited journals include the “Journal of Applied Psychology” and “Journal of Vocational Behavior,” addressing gender role differences, gender equality, and work-family satisfaction. Notable references include V Braun’s 2021 introduction to thematic analysis methods for semi-structured interviews in WFB research and JF Hair’s significant 2018 work on PLS-SEM, which is widely applied despite not being directly related to WFB themes. Seminal references have identified key subfields that cover individual factors (e.g., gender division), environmental influences (e.g., COVID-19), organizational support strategies (e.g., family supportive supervisor behavior), family support initiatives, childcare subsidies, relevant research methodologies, and theoretical foundations.
4. Emerging hotspots and prospects in this field: WFB spans interdisciplinary areas, such as public health, psychology, family dynamics, and social sciences. Future research should focus on category co-occurrence related to family, occupation, perspective, and theory. Key topics include gender differences in WFC, satisfaction evaluation applications in WFB, childcare pressures, mental health challenges, and time management for women to balance work and family; the low fertility rate phenomenon is linked to the pursuit of WFB amid higher education, obstacles to WFB and WLB, and supportive measures to address these issues.

Furthermore, recent studies have focused on the moderating role of WFB and WFE, indicating new research directions. Future research will focus on interdisciplinary collaboration among family studies, management, and applied psychology to explore harmonious labor relations through organizational management while prioritizing employee mental health. This involved a comparative analysis of psychological enrichment in separated or divorced versus intact families, aiming to identify optimal WFB solutions for different family structures. Second, broader themes examine not only gender differences in household labor division, parenting roles, and career advancement but also strategies for achieving WFE to create a balanced lifestyle. Third, emerging theories and analytical methods, such as positioning theory, the technology acceptance model, and life-history theory, will be applied in WFB research. There will be continued integration of qualitative and quantitative analyses along with methodologies from other fields (e.g., fsQCA, coupling coordination, and gray prediction). Moreover, the importance of cross-regional and cross-cultural collaborative research is also increasing. Countries with high WFB satisfaction (e.g., Canada and the Netherlands) can provide practical insights for those lacking it (e.g., China and South Korea). Scholars should closely monitor social phenomena trends to enrich WFB research based on real-world contexts.

### 5.2. Contributions and innovations

This research introduces the following innovative factors:

1. Comprehensive analysis: Using bibliometric methods, this study performed statistical, collaborative, co-citation, and co-occurrence analyses of the existing WFB research. It emphasized the diversity in WFB studies across 4 main questions, addressing a gap in comprehensive field analysis.
2. Research framework: A panoramic knowledge framework for WFB was established, illustrating its structure and key topics, while clarifying the context.
3. Future directions: The study identified key hot topics and future trends in WFB, enriching the knowledge system and guiding scholars in selecting related research areas.

### 5.3. Limitations and future work

The limitations of this study include:

1. Time selection constraint: The literature analyzed covers January 1, 2000 to December 31, 2024, excluding earlier data and recent articles from 2025. This may overlook the foundational and current studies.
2. Language constraints: Only English publications were included, potentially missing high-quality articles and notable non-English authors.
3. Limitations of bibliometric methods: Focusing on highly cited recent articles may underestimate less-cited research; work by independent authors and institutions might be overlooked; and content with low co-citation and co-occurrence may not receive attention. Additionally, the data sources were limited to SCI, SSCI, and AHCI, which could introduce bias by omitting important literature in other databases.

Future research should employ a wider range of publication databases to analyze the latest articles and utilize various bibliometric software packages. Removing language restrictions is also crucial for incorporating non-English content to obtain more comprehensive results.

## Author contributions

**Conceptualization:** Yan Yan.

**Data curation:** Yan Yan.

**Formal analysis:** Yan Yan.

**Funding acquisition:** Yan Yan.

**Investigation:** Xiaohan Zhang, Chengxin Ye.

**Methodology:** Xiaohan Zhang.

**Project administration:** Xiaohan Zhang.

**Resources:** Chengxin Ye.

**Software:** Xiaohan Zhang.

**Supervision:** Yan Yan.

**Validation:** Xiaohan Zhang.

**Visualization:** Yan Yan.

**Writing – original draft:** Yan Yan, Xiaohan Zhang, Chengxin Ye.

**Writing – review & editing:** Jianyi Li, Juan Gao, Yuqing Geng.
